# Effects of intensive lifestyle changes on the progression of mild cognitive impairment or early dementia due to Alzheimer’s disease: a randomized, controlled clinical trial

**DOI:** 10.1186/s13195-024-01482-z

**Published:** 2024-06-07

**Authors:** Dean Ornish, Catherine Madison, Miia Kivipelto, Colleen Kemp, Charles E. McCulloch, Douglas Galasko, Jon Artz, Dorene Rentz, Jue Lin, Kim Norman, Anne Ornish, Sarah Tranter, Nancy DeLamarter, Noel Wingers, Carra Richling, Rima Kaddurah-Daouk, Rob Knight, Daniel McDonald, Lucas Patel, Eric Verdin, Rudolph E. Tanzi, Steven E. Arnold

**Affiliations:** 1 Preventive Medicine Research Institute, 900 Bridgeway, Sausalito, CA USA; 2 University of California, San Francisco and University of California, San Diego, USA; 3 Ray Dolby Brain Health Center, California Pacific Medical Center, San Francisco, CA USA; 4 Division of Clinical Geriatrics, Department of Neurobiology, Care Sciences and Society, Karolinska Institute, Karolinska vägen 37 A, SE-171 64 Solna, Sweden; 5 Theme Inflammation and Aging, Karolinska University Hospital, Karolinska vägen 37 A, SE-171 64 Stockholm, Solna Sweden; 6 The Ageing Epidemiology (AGE) Research Unit, School of Public Health, Imperial College London, St Mary’s Hospital, Norfolk Place, London, W2 1PG United Kingdom; 7 Institute of Public Health and Clinical Nutrition, University of Eastern Finland, Yliopistonranta 8, 70210 Kuopio, Finland; 8 Clinical Services, Preventive Medicine Research Institute, Bridgeway, Sausalito, CA 900 USA; 9 Division of Biostatistics, Department of Epidemiology & Biostatistics, UCSF, San Francisco, CA USA; 10 Neurosciences, University of California, San Diego, CA USA; 11 Clinical Neurology, School of Medicine, University of Nevada, Reno, USA; 12 Renown Health Institute of Neurosciences, Reno, NV USA; 13 Harvard Medical School, Boston, MA USA; 14 Center for Alzheimer Research and Treatment, Boston, MA USA; 15 Mass General Brigham Alzheimer Disease Research Center, Boston, MA USA; 16 Elizabeth Blackburn Lab, UCSF, San Francisco, CA USA; 17 UCSF, San Francisco, CA USA; 18 Departments of Medicine and Psychiatry, Duke University Medical Center and Member, Duke Institute of Brain Sciences, Durham, NC USA; 19 Department of Pediatrics; Department of Computer Science & Engineering; Department of Bioengineering; Center for Microbiome Innovation, Halıcıoğlu Data Science Institute, University of California, San Diego, La Jolla, CA USA; 20 Department of Pediatrics and Scientific Director, American Gut Project and The Microsetta Initiative, University of California San Diego, La Jolla, CA USA; 21 Bioinformatics and Systems Biology Program; Rob Knight Lab; Medical Scientist Training Program, University of California, San Diego, La Jolla, CA USA; 22 Buck Institute for Research on Aging, San Francisco, CA USA; 23 University of California, San Francisco, CA USA; 24 Genetics and Aging Research Unit, Boston, MA USA; 25 McCance Center for Brain Health, Boston, MA USA; 26 Massachusetts General Hospital, Boston, MA USA; 27 Interdisciplinary Brain Center, Massachusetts General Hospital, Boston, MA USA

**Keywords:** Alzheimer’s, Diet, Nutrition, Exercise, Stress management, Lifestyle medicine, Social support

## Abstract

**Background:**

Evidence links lifestyle factors with Alzheimer’s disease (AD). We report the first randomized, controlled clinical trial to determine if intensive lifestyle changes may beneficially affect the progression of mild cognitive impairment (MCI) or early dementia due to AD.

**Methods:**

A 1:1 multicenter randomized controlled phase 2 trial, ages 45-90 with MCI or early dementia due to AD and a Montreal Cognitive Assessment (MoCA) score of 18 or higher. The primary outcome measures were changes in cognition and function tests: Clinical Global Impression of Change (CGIC), Alzheimer’s Disease Assessment Scale (ADAS-Cog), Clinical Dementia Rating–Sum of Boxes (CDR-SB), and Clinical Dementia Rating Global (CDR-G) after 20 weeks of an intensive multidomain lifestyle intervention compared to a wait-list usual care control group. ADAS-Cog, CDR-SB, and CDR-Global scales were compared using a Mann-Whitney-Wilcoxon rank-sum test, and CGIC was compared using Fisher’s exact test. Secondary outcomes included plasma Aβ42/40 ratio, other biomarkers, and correlating lifestyle with the degree of change in these measures.

**Results:**

Fifty-one AD patients enrolled, mean age 73.5. No significant differences in any measures at baseline. Only two patients withdrew. All patients had plasma Aβ42/40 ratios <0.0672 at baseline, strongly supporting AD diagnosis. After 20 weeks, significant between-group differences in the CGIC (*p*= 0.001), CDR-SB (*p*= 0.032), and CDR Global (*p*= 0.037) tests and borderline significance in the ADAS-Cog test (*p*= 0.053). CGIC, CDR Global, and ADAS-Cog showed improvement in cognition and function and CDR-SB showed significantly less progression, compared to the control group which worsened in all four measures. Aβ42/40 ratio increased in the intervention group and decreased in the control group (*p* = 0.003). There was a significant correlation between lifestyle and both cognitive function and the plasma Aβ42/40 ratio. The microbiome improved only in the intervention group (*p* <0.0001).

**Conclusions:**

Comprehensive lifestyle changes may significantly improve cognition and function after 20 weeks in many patients with MCI or early dementia due to AD.

**Trial registration:**

Approved by Western Institutional Review Board on 12/31/2017 (#20172897) and by Institutional Review Boards of all sites. This study was registered retrospectively with clinicaltrials.gov on October 8, 2020 (NCT04606420, ID: 20172897).

**Supplementary Information:**

The online version contains supplementary material available at 10.1186/s13195-024-01482-z.

## Background

Increasing evidence links lifestyle factors with the onset and progression of dementia, including AD. These include unhealthful diets, being sedentary, emotional stress, and social isolation.

For example, a *Lancet* commission on dementia prevention, intervention, and care listed 12 potentially modifiable risk factors that together account for an estimated 40% of the global burden of dementia [1]. Many of these factors (e.g., hypertension, smoking, depression, type 2 diabetes, obesity, physical inactivity, and social isolation) are also risk factors for coronary heart disease and other chronic illnesses because they share many of the same underlying biological mechanisms. These include chronic inflammation, oxidative stress, insulin resistance, telomere shortening, sympathetic nervous system hyperactivity, and others [2]. A recent study reported that the association of lifestyle with cognition is mostly independent of brain pathology, though a part, estimated to be only 12%, was through β-amyloid [3].

In one large prospective study of adults 65 or older in Chicago, the risk of developing AD was 38% lower in those eating high vs low amounts of vegetables and 60% lower in those consuming omega-3 fatty acids at least once/week, [4] whereas consuming saturated fat and trans fats more than doubled the risk of developing AD [5].A systematic review and meta-analysis of 243 observational prospective studies and 153 randomized controlled trials found a similar relationship between these and similar risk factors and the onset of AD [6].

The multifactorial etiology and heterogeneity of AD suggest that multidomain lifestyle interventions may be more effective than single-domain ones for reducing the risk of dementia, and that more intensive multimodal lifestyle interventions may be more efficacious than moderate ones at preventing dementia [7].

For example, in the Finnish Geriatric Intervention Study (FINGER) study, a RCT of men and women 60-77 in age with Cardiovascular Risk Factors, Aging, and Incidence of Dementia (CAIDE) dementia risk scores of at least 6 points and cognition at mean or slightly lower, a multimodal intervention of diet, exercise, cognitive training, vascular risk monitoring maintained cognitive function after 2 years in older adults at increased risk of dementia [8]. After 24 months, global cognition in the FINGER intervention group was 25% higher than in the control group which declined. Moreover, the FINGER intervention was equally beneficial regardless of several demographic and socioeconomic risk factors [9] and apolipoprotein E (APOE) ε4 status [10].

The FINGER lifestyle intervention also resulted in a 13-20% reduction in rates of cardiovascular disease events (stroke, transient ischemic attack, or coronary), providing more evidence that “what’s good for the heart is good for the brain”(and vice versa) [11]. Other large-scale multidomain intervention studies to determine if this intervention can help prevent dementia are being conducted or planned in over 60 countries worldwide, as part of the World-Wide FINGERS network, including the POINTER study in the U.S. [12, 13].

More recently, a similar dementia prevention-oriented RCT showed that a 2-year personalized multidomain intervention led to modest improvements in cognition and dementia risk factors in those at risk for (but not diagnosed with) dementia and AD [14].

All these studies showed that lifestyle changes may help prevent dementia. The study we are reporting here is the first randomized, controlled clinical trial to test whether intensive lifestyle changes may beneficially affect those already diagnosed with mild cognitive impairment (MCI) or early dementia due to AD.

In two earlier RCTs, we found that the same multimodal lifestyle intervention described in this article resulted in regression of coronary atherosclerosis as measured by quantitative coronary arteriography [15] and ventricular function, [16] improvements in myocardial perfusion as measured by cardiac PET scans, and 2.5 times fewer cardiac events after five years, all of which were statistically significant [17]. Until then, it was believed that coronary heart disease progression could only be slowed, not stopped or reversed, similar to how MCI or early dementia due to AD are viewed today.

Since AD and coronary heart disease share many of the same risk factors and biological mechanisms, and since moderate multimodal lifestyle changes may help prevent AD, [18] we hypothesized that a more intensive multimodal intervention proven to often reverse the progression of coronary heart disease and some other chronic diseases may also beneficially affect the progression of MCI or early dementia due to AD.

We report here results of a randomized controlled trial to determine if the progression of MCI or early dementia due to AD may be slowed, stopped, or perhaps even reversed by a comprehensive, multimodal, intensive lifestyle intervention after 20 weeks when compared to a usual-care randomized control group. This lifestyle intervention includes (1) a whole foods, minimally processed plant-based diet low in harmful fats and low in refined carbohydrates and sweeteners with selected supplements; (2) moderate exercise; (3) stress management techniques; and (4) support groups.

This intensive multimodal lifestyle modification RCT sought to address the following questions:

1. Can the specified multimodal intensive lifestyle changes beneficially affect the progression of MCI or early dementia due to AD as measured by the AD Assessment Scale–Cognitive Subscale (ADAS-Cog), CGIC (Clinical Global Impression of Change), CDR-SB (Clinical Dementia Rating Sum of Boxes), and CDR-G (Clinical Dementia Rating Global) testing?
2. Is there a significant correlation between the degree of lifestyle change and the degree of change in these measures of cognition and function?
3. Is there a significant correlation between the degree of lifestyle change and the degree of change in selected biomarkers (e.g., the plasma Aβ42/40 ratio)?

## Methods

### Participants and methods

This study was a 1:1 multi-center RCT during the first 20 weeks of the study, and these findings are reported here. Patients who met the clinical trial inclusion criteria were enrolled between September 2018 and June 2022.

Participants were enrolled who met the following inclusion criteria:

- Male or female, ages 45 to 90
- Current diagnosis of MCI or early dementia due to AD process, with a MoCA score of 18 or higher (National Institute on Aging–Alzheimer’s Association McKhann and Albert 2011 criteria) [19, 20]
- Physician shared this diagnosis with the patient and approved their participation in this clinical trial
- Willingness and ability to participate in all aspects of the intervention
- Availability of spouse or caregiver to provide collateral information and assist with study adherence

Patients were excluded if they had any of the following:

- Moderate or severe dementia
- Physical disability that precludes regular exercise
- Evidence for other primary causes of neurodegeneration or dementia, e.g., significant cerebrovascular disease (whose primary cause of dementia was vascular in origin), Lewy Body disease, Parkinson's disease, FTD
- Significant ongoing psychiatric or substance abuse problems

Fifty-one participants with MCI or early-stage dementia due to AD who met these inclusion criteria were enrolled between September 2018 and June 2022 and underwent baseline testing. 26 of the enrolled participants were randomly assigned to an intervention group that received the multimodal lifestyle intervention for 20 weeks and 25 participants were randomly assigned to a usual habits and care control group that was asked not to make any lifestyle changes for 20 weeks, after which they would be offered the intervention. Patients in both groups received standard of care treatment managed by their own neurologist.

The intervention group received the lifestyle program for 20 weeks (initially in person, then via synchronous Zoom after March 2020 due to COVID-19). Two participants who did not want to continue these lifestyle changes withdrew during this time, both in the intervention group (one male, one female). Participants in both groups completed a follow-up visit at 20 weeks, where clinical and cognitive assessments were completed. Data were analyzed comparing the baseline and 20 week assessments between the groups.

In a drug trial, access to an investigational new drug can be restricted from participants in a randomized control group. However, we learned in our prior clinical trials of this lifestyle intervention with other diseases that it is often difficult to persuade participants who are randomly assigned to a usual-care control group to refrain from making these lifestyle changes for more than 20 weeks, which is why this time duration was chosen. If participants in both groups made similar lifestyle changes, then it would not be possible to show differences between the groups. Therefore, to encourage participants randomly assigned to the control group not to make lifestyle changes during the first 20 weeks, we offered to provide them the same lifestyle program at no cost to them for 20 weeks after being in the usual-care control group and tested after 20 weeks.

We initially planned to enroll 100 patients into this study based on power calculations of possible differences between groups in cognition and function after 20 weeks. However, due to challenges in recruiting patients, especially with the COVID-19 emergency and that many pharma trials began recruiting patients with similar criteria, it took longer to enroll patients than initially planned [21]. Because of this, we terminated recruitment after 51 patients were enrolled. This decision was based only on recruitment issues and limited funding, without reviewing the data at that time.

Patients were recruited from advertisements, presentations at neurology meetings, referrals from diverse groups of neurologists and other physicians, and a search of an online database of patients at UCSF. We put a special emphasis on recruiting diverse patients, although we were less successful in doing so than we hoped (Table 1).

### Oversight

This clinical trial was approved by the Western Institutional Review Board on 12/31/2017 (approval number: 20172897) and all participants and their study partners provided written informed consent. The trial protocol was also approved by the appropriate Institutional Review Board of all participating sites, and all subjects provided informed consent. Due to the COVID-19 emergency, planned MRI and amyloid PET scans were no longer feasible, and the number of cognition and function tests was decreased. An initial inclusion criterion of “current diagnosis of mild to moderate dementia due to AD (McKhann et al., 2011)” was further clarified to include a MoCA score of 18 or higher. This study was registered with clinicaltrials.gov on October 8, 2020 (NCT04606420, Unique Protocol ID: 20172897) retrospectively due to an administrative error. None of the sponsors who provided funding for this study participated in its design, conduct, management, or reporting of the results. Those providing the lifestyle intervention were separate from those performing testing and from those collecting and analyzing the data, who were blinded to group assignment. All authors contributed to manuscript draft revisions, provided critical comment, and approved submission for publication.

Any modifications in the protocol were approved in advance and in writing by the senior biostatistician (Charles McCulloch PhD) or the senior expert neuropsychologist (Dorene Rentz PsyD), and subsequently approved by the WIRB.

### Setting

Patients were initially recruited only from the San Francisco Bay area beginning October 2018 and met in person until February 2020 when the COVID-19 pandemic began. Subsequently, this multimodal lifestyle intervention was offered to patients at home in real time via Zoom.

Offering this intervention virtually provided an opportunity to recruit patients from multiple sites, including the Massachusetts General Hospital/Harvard Medical School, Boston, MA; the University of California, San Diego; and Renown Regional Medical Center, Reno, NV, as well as with neurologists in the San Francisco Bay Area. These participants were recruited and tested locally at each site and the intervention was provided via Zoom and foods were sent directly to their home.

### Patient recruitment

This is described in the Supplemental Materials section.

### Intensive multimodal lifestyle intervention

Each patient received a copy of a book which describes this lifestyle medicine intervention for other chronic diseases. [2]

#### Diet

A whole foods minimally-processed plant-based (vegan) diet, high in complex carbohydrates (predominantly fruits, vegetables, whole grains, legumes, soy products, seeds and nuts) and especially low in harmful fats, sweeteners and refined carbohydrates. It was approximately 14-18% of calories as total fat, 16-18% protein, and 63-68% mostly complex carbohydrates. Calories were unrestricted. Those with higher caloric needs were given extra portions.

To assure the high adherence and standardization required to adequately test the hypothesis, 21 meals/week and snacks plus the daily supplements listed below were provided throughout the 40 weeks of this intervention to each study participant and his or her spouse or study partner at no cost to them. Twice/week, we overnight shipped to each patient as well as to their spouse or study partner three meals plus two snacks per day that met the nutritional guidelines as well as the prescribed nutritional supplements.

We asked participants to consume only the food and nutritional supplements we sent to them and no other foods. We reasoned that if adherence to the diet and lifestyle intervention was high, whatever outcomes we measured would be of interest. That is, if patients in the intervention group were adherent but showed no significant benefits, that would be a disappointing but an important finding. If they showed improvement, that would also be an important finding. But if they did not follow the lifestyle intervention sufficiently, then we would not have been able to adequately test the hypotheses.

##### Exercise

Aerobic (e.g., walking) at least 30 minutes/day and mild strength training exercises at least three times per week from an exercise physiologist in person or with virtual sessions. Patients were given a personalized exercise prescription based on age and fitness level. All sessions were overseen by a registered nurse.

##### Stress management

Meditation, gentle yoga-based poses, stretching, progressive relaxation, breathing exercises, and imagery for a total of one hour per day, supervised by a certified stress management specialist. The purpose of each technique was to increase the patient’s sense of relaxation, concentration, and awareness. They were also given access to online meditations. Patients had the option of using flashing-light glasses at a theta frequency of 7.83 Hz plus soothing music as an aid to meditation and insomnia [22]. They were also encouraged to get adequate sleep.

##### Group support

Participants and their spouses/study partners participated in a support group one hour/session, three days/week, supervised by a licensed mental health professional in a supportive, safe environment to increase emotional support and community as well as communication skills and strategies for maintaining adherence to the program. They also received a book with memory exercises used periodically during group sessions [23].

To reinforce this lifestyle intervention, each patient and their spouse or study partner met three times/week, four hours/session via Zoom:^2^

- one hour of supervised exercise (aerobic + strength training)
- one hour of stress management practices (stretching, breathing, meditation, imagery)
- one hour of a support group
- one hour lecture on lifestyle

Additional optional exercise and stress management classes were provided.

### Supplements

- Omega-3 fatty acids with Curcumin (1680 mg omega-3 & 800 mg Curcumin, Nordic Naturals ProOmega CRP, 4 capsules/day). Omega-3 fatty acids: In those age 65 or older, those consuming omega-3 fatty acids once/week or more had a 60% lower risk of developing AD, and total intake of n-3 polyunsaturated fatty acids was associated with reduced risk of Alzheimer disease [24]. Curcumin targets inflammatory and antioxidant pathways as well as (directly) amyloid aggregation, [25] although there may be problems with bioavailability and crossing the blood-brain barrier [26].
- Multivitamin and Minerals (Solgar VM-75 without iron, 1 tablet/day). Combinatorial formulations demonstrate improvement in cognitive performance and the behavioral difficulties that accompany AD [27].
- Coenzyme Q10 (200 mg, Nordic Naturals, 2 soft gels/day). CoQ10. May reduce mitochondrial impairment in AD [28].
- Vitamin C (1 gram, Solgar, 1 tablet/day): Maintaining healthy vitamin C levels may have a protective function against age-related cognitive decline and AD [29].
- Vitamin B12 (500 mcg, Solgar, 1 tablet/day): B12 hypovitaminosis is linked to the development of AD pathology [30].
- Magnesium L-Threonate (Mg) (144 mg, Magtein, 2 tablets/day). A meta-analysis found that Mg deficiency may be a risk factor of AD and Mg supplementation may be an adjunctive treatment for AD [31].
- Hericium erinaceus (Lion’s Mane, Stamets Host Defense, 2 grams/day): Lion’s mane may produce significant improvements in cognition and function in healthy people over 50 [32] and in MCI patients compared to placebo [33].
- Super Bifido Plus Probiotic (Flora, 1 tablet/day). A meta-analysis suggests that probiotics may benefit AD patients [34].

### Primary outcome measures: cognition and function testing

Four tests were used to assess changes in cognition and function in these patients. These are standard measures of cognition and function included in many FDA drug trials: ADAS-Cog; Clinical Global Impression of Change (CGIC); Clinical Dementia Rating Sum of Boxes (CDR-SB); Clinical Dementia Rating Global (CDR Global). All cognition and function raters were trained psychometrists with experience in administering these tests in clinical trials. Efforts were made to have the same person perform cognitive testing at each visit to reduce inter-observer variability. Those doing ADAS-Cog assessments were certified raters and tested patients in person. The CGIC and CDR tests were administered for all patients via Zoom by different raters than the ADAS-cog. Also, raters were blind to treatment arm to the degree possible.

### Secondary outcome measures: biomarkers and microbiome

These are described in the Supplemental Materials section. These include blood-based biomarkers (such as the plasma Aβ42/40 ratio) and microbiome taxa (organisms).

### Statistical methods

These are described in the Supplemental Materials section.

## Results

**Fig. 1:**
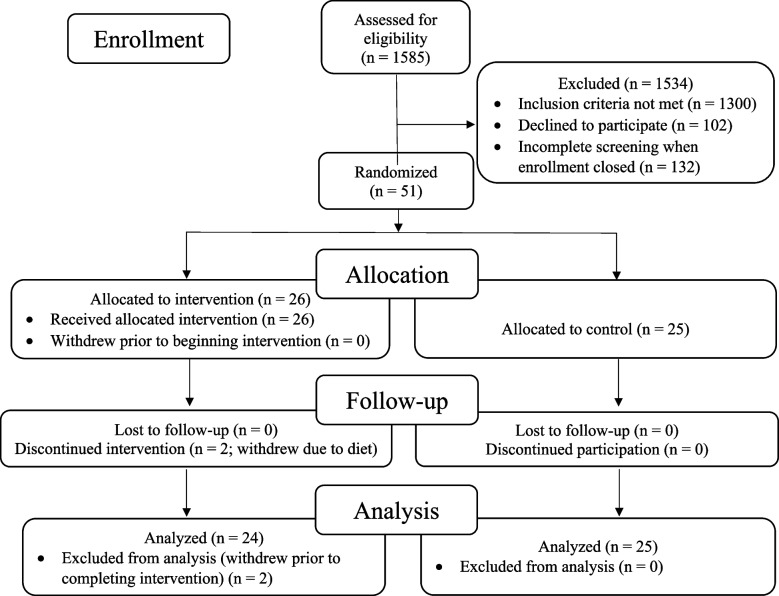
CONSORT flowchart: patients, demographics, and enrollment

The recruitment effort for this trial lasted from 01/23/2018 to 6/16/2022. The most effective recruitment method was referral from the subjects’ physician or healthcare provider. Additional recruitment efforts included advertising in print and digital media; speaking to community groups; mentioning the study during podcast and radio interviews; collaborating with research institutions that provide dementia diagnosis and treatment; and contracting a clinical trials recruitment service (Linea). A total of 1585 people contacted us; of these, 1300 did not meet the inclusion criteria, 102 declined participation, and 132 were screening incomplete when enrollment closed, resulting in the enrollment of 51 participants (Fig. 1).

The remaining 51 patients were randomized to an intervention group (26 patients) that received the lifestyle intervention for 20 weeks or to a usual-care control group (25 patients) that was asked not to make any lifestyle changes. Two patients in the intervention group withdrew during the intervention because they did not want to continue the diet and lifestyle changes. No patients in the control group withdrew prior to 20-week testing. Analyses were performed on the remaining 49 patients. No patients were lost to follow-up.

All of these 49 patients had plasma Aβ42/40 ratios <0.089 (all were <0.0672), strongly supporting the diagnosis of Alzheimer’s disease [35].

At baseline, there were no statistically significant differences between the intervention group and the randomized control group in any measures, including demographic characteristics, cognitive function measures, or biomarkers (Table 1 and Table 2).

**Table 1:** Categorical baseline values

	**Baseline Values**				
	**Control**		**Intervention**		
**Characteristic**	**n**	**Percent**	**N**	**Percent**	***p-value***
**Sex**					
Male	13	42.9	19	73.1	0.153
Female	12	48	7	26.9	
**Marital Status**					
Married	21	84	18	69.2	0.504
Single	3	12	7	26.9	
Widowed	1	4	1	3.85	
**Living situation** Lives with study partner	22	88	20	76.9	0.465
Lives alone	3	12	6	23.1	
**Cognitive tests**					
**CGIC:**					0.26
2 - Borderline Ill	5	20	8	33.3	
3 - Mildly Ill	11	44	9	37.5	
4 - Moderately Ill	7	28	3	12.5	
5 - Markedly Ill	1	4	4	16.7	
6 - Severely Ill	1	4	0	0	
**CDR Global**					
0.5	17	68	15	62.5	0.769
1	8	32	9	37.5	
**Race:**					
African-American	1	4	1	3.85	1
Asian	0	0	1	3.85	
Hispanic	1	4	1	3.85	
Caucasian	23	92	23	88.46	
**APO**ε** status**					
2/3	1	4	0	0	1
2/4	0	0	0	0	
3/3	9	36	10	38.5	
3/4	11	44	11	42.3	
4/4	4	16	5	19.2	

**Table 2:** Numeric baseline values

	**Baseline Values**				
	**Control**		**Intervention**		
**Characteristic**	**Mean**	**SD**	**Mean**	**SD**	***p-value***
Age, mean, years	75	7.1	72	8.5	0.25
Education level mean, ISCE	6.52	1.5	6.58	1	0.724
**Cognitive tests**					
**ADAS-cog**	21.25	6.46	21.44	5.92	0.886
**CDR-SB**	3.34	1.84	3.27	1.73	0.925
**Baseline Biomarkers**					
Plasma Aβ42/40 ratio	0.048	0.0098	0.047	0.0089	0.83
Phosphorylated Tau181	45.32	16.34	43.57	14.22	0.959
Insulin	9.5	9.85	8.68	5.79	0.834
HgbA1C	5.47	0.38	5.38	0.37	0.856
LDL cholesterol	1.91	0.34	1.93	0.51	0.443
C-peptide	2005.27	1661.32	1907.38	866.23	0.814
Glycoprotein acetylation (GlycA)	0.843	0.092	0.891	0.117	0.097
Glial Fibrillary Acidic Protein	250.27	104.89	229.68	93.72	0.48
C-reactive Protein (CRP)	2.02	2.2	2	3.05	0.894

### Cognition and function testing: primary analysis

Results after 20 weeks of a multimodal intensive lifestyle intervention in all patients showed overall statistically significant differences between the intervention group and the randomized control group in cognition and function in the CGIC (*p*= 0.001), CDR-SB (*p*= 0.032), and CDR Global (*p*= 0.037) tests and of borderline significance in the ADAS-Cog test (*p*= 0.053, Table 3). Three of these measures (CGIC, CDR Global, ADAS-Cog) showed improvement in cognition and function in the intervention group and worsening in the control group, and one test (CDR-SB) showed significantly less progression when compared to the randomized control group, which worsened in all four of these measures.

**Table 3:** Cognition and function test results

	**0-week**		**20-week**		**Change over 20 weeks**		
**Cognitive test**	**Control**	**Intervention**	**Control**	**Intervention**	**Control**	**Intervention**	***p-value***
ADAS-cog	21.25	21.55	22.16	20.54	0.91	-1.01	0.053
CDR-SB	3.34	3.27	3.86	3.35	0.52	0.08	0.032
CDR-Global	0.66	0.69	0.74	0.65	0.08	-0.04	0.037
CGIC baseline							
2 - Borderline Ill	5	8					
3 - Mildly Ill	11	9					
4 - Moderately Ill	7	3					
5 - Markedly Ill	1	4					
6 - Severely Ill	1	0					
CGIC changes							0.001
3 – Min Improved			0	10			
4 - Unchanged			8	7			
5 - Min Worsening			14	7			
6 - Mod Worsening			3	0			

PRIMARY ANALYSIS (with outlier included), Table 3:

1. CGIC (Clinical Global Impression of Change)

These scores improved in the intervention group and worsened in the control group.

(Fisher’s exact *p*-value = 0.001). 10 people in the intervention group showed improvement compared to none in the control group. 7 people in the intervention group and 8 people in the control group were unchanged. 7 people in the intervention group showed minimal worsening compared to 14 in the control group. None in the intervention group showed moderate worsening compared to 3 in the control group.

2. CDR-Global (Clinical Dementia Rating-Global)

**Fig. 2:**
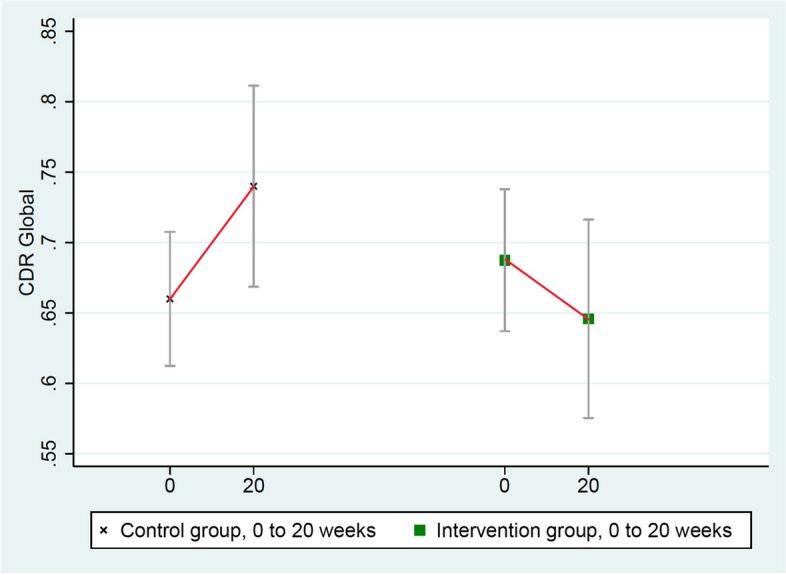
Changes in CDR-Global (lower = improved)

These scores improved in the intervention group (from 0.69 to 0.65) and worsened in the randomized control group (from 0.66 to 0.74), mean difference = 0.12, *p* = 0.037 (Table 3 and Fig. 2).

3. ADAS-Cog (Alzheimer’s Disease Assessment Scale)

**Fig. 3:**
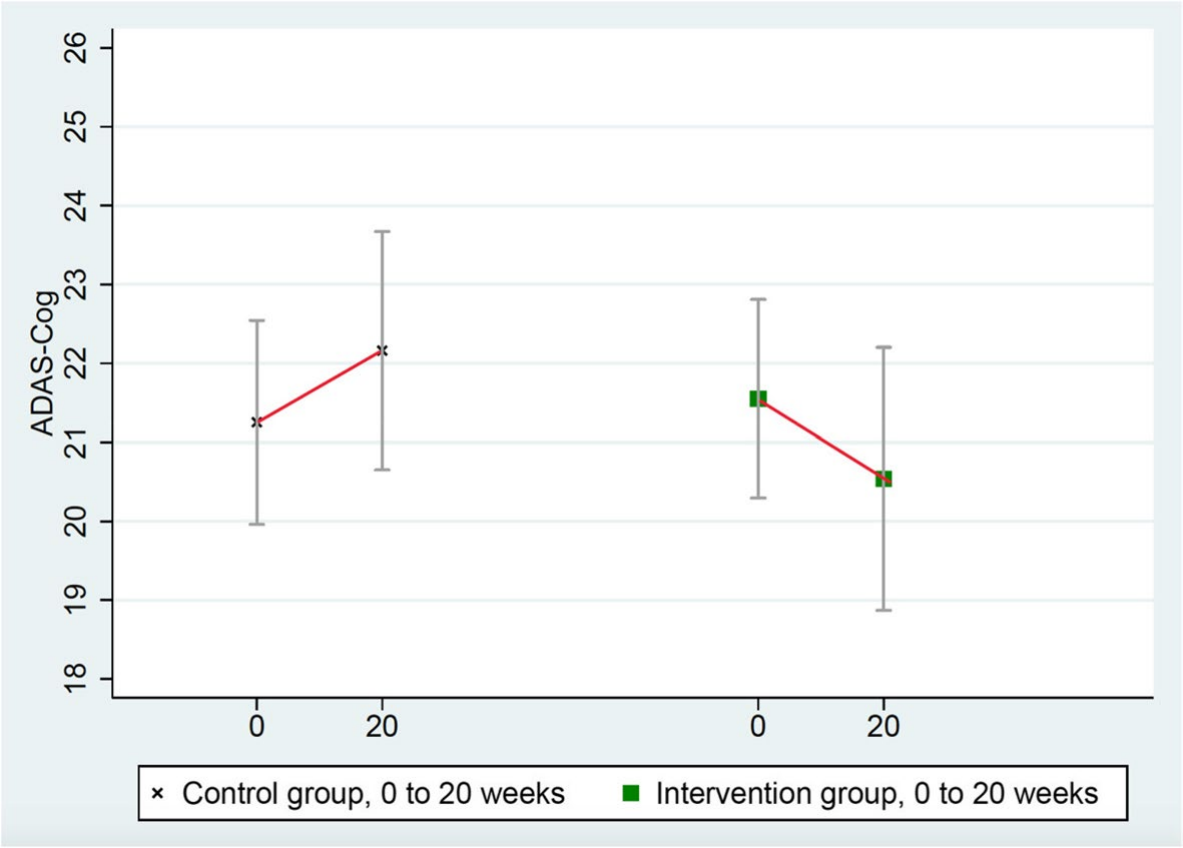
Changes in ADAS-Cog (lower = improved)

These scores improved in the intervention group (from 21.551 to 20.536) and worsened in the randomized control group (from 21.252 to 22.160), mean group difference of change = 1.923 points, *p* = 0.053 (Table 3 and Fig. 3). (ADAS-Cog testing in one intervention group patient was not administered properly so it was excluded.)

4. CDR-SB (Clinical Dementia Rating Sum of Boxes)

**Fig. 4:**
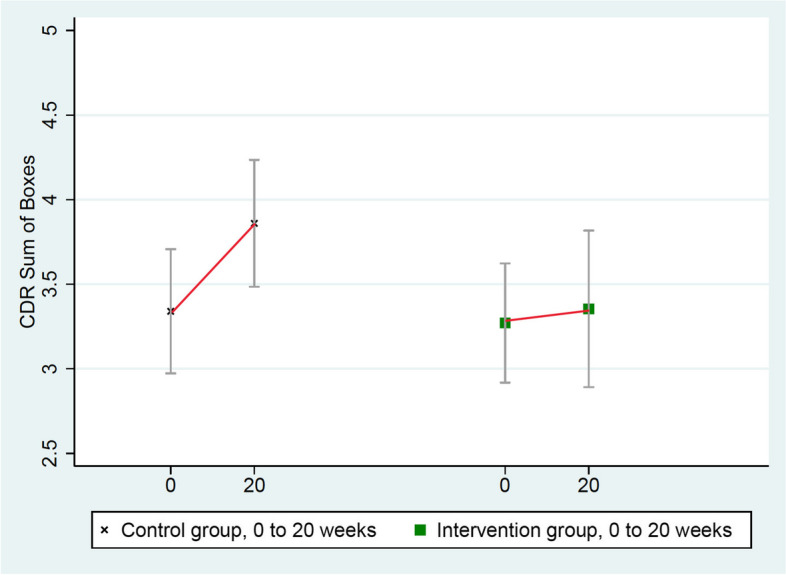
Changes in CDR-SB (lower = improved)

These scores worsened significantly more in the control group (from 3.34 to 3.86) than in the intervention group (from 3.27 to 3.35), mean group difference = 0.44, *p* = 0.032 (Table 3 and Fig. 4).

There were no significant differences in depression scores as measured by PHQ-9 between the intervention and control groups.

### Secondary sensitivity analyses

One patient in the intervention group was a clear statistical outlier in his cognitive function testing based on standard mathematical definitions (none was an outlier in the control group) [36]. Therefore, this patient’s data were excluded in a secondary sensitivity analysis. These results showed statistically significant differences in all four of these measures of cognition and function (Table 4). Three measures (ADAS-Cog, CGIC, and CDR Global) showed significant improvement in cognition and function and one (CDR-SB) showed significantly less worsening when compared to the randomized control group, which worsened in all four of these measures.

**Table 4:** Cognition and function data with sensitivity analysis (with outlier excluded):

	**0-week**		**20-week**		**Change over 20 weeks**		
**Cognitive test**	**Control**	**Intervention**	**Control**	**Intervention**	**Control**	**Intervention**	***p-value***
ADAS-cog	21.25	21.62	22.16	20.03	0.91	-1.59	0.028
CDR-SB	3.34	3.22	3.86	3.33	0.52	0.11	0.046
CDR-Global	0.66	0.67	0.74	0.63	0.08	-0.04	0.037
CGI-ds							
2 - Borderline Ill	5	8					
3 - Mildly Ill	11	8					
4 - Moderately Ill	7	3					
5 - Markedly Ill	1	4					
6 - Severely Ill	1	0					
CGI-c							0.001
3 - Minimal Improvement			0	10			
4 - Unchanged			8	6			
5 - Minimal Worsening			14	7			
6 - Moderate Worsening			3	0			

### Sensitivity analysis (with outlier excluded)

There were no significant differences in depression scores as measured by PHQ-9 between the intervention and control groups in either analysis.

A reason why this patient might have been a statistical outlier is that he reported intense situational stress before his testing. As a second sensitivity analysis, this same outlier patient was retested when he was calmer, and all four measures (ADAS-Cog, CGIC, CDR Global, and CDR-SB) showed significant improvement in cognition and function, whereas the randomized control group worsened in all four of these measures.

### Biomarker results

We selected biomarkers that have a known role in the pathophysiology of AD (Table 5). Of note is that the plasma Aβ42/40 ratio increased in the intervention group but decreased in the randomized control group (*p* = 0.003, two-tailed).

**Table 5:** Biomarker results

	**0-week**		**20-week**		**Change over 20 weeks**		
**Biomarker**	**Control**	**Intervention**	**Control**	**Intervention**	**Control**	**Intervention**	***p-value***
Plasma AB42/40 Ratio	0.048	0.047	0.044	0.049	-0.004	0.003	0.003
Phosphorylated Tau181 (pTau181)	45.32	43.57	50.72	43.74	5.40	0.17	0.209
Insulin	9.50	8.68	8.21	6.77	-1.30	-1.92	0.048
Beta-Hydroxybutyrate (ketones)	0.11	0.09	0.06	0.11	-0.05	0.02	0.021
LDL cholesterol	1.91	1.93	1.95	1.32	0.04	-0.61	<0.001
Glial Fibrillary Acidic Protein (GFAP)	250.27	229.68	282.69	232.85	32.42	3.16	0.199
C-reactive Protein (CRP)	2.02	2.00	1.75	0.95	-0.27	-1.05	0.373
Serum amyloid A (SAA)	6.42	5.79	6.30	4.14	-0.12	-1.65	0.074
Glycoprotein acetylation (GlycA)	0.843	0.891	0.83	0.81	-0.01	-0.08	0.005
Telomere length	0.774	0.799	0.776	0.814	0.002	0.015	0.287

## Correlation of lifestyle index and cognitive function

In the current clinical trial, despite the inherent limitations of self-reported data, we found statistically significant correlations between the degree of lifestyle change (from baseline to 20 weeks) and the degree of change in three of four measures of cognition and function as well as correlations between the adherence to desired lifestyle changes at just the 20-week timepoint and the degree of change in two of the four measures of cognition and function and borderline significance in the fourth measure.

### ADAS-Cog

Correlation with lifestyle at 20 weeks: *p* = 0.052; correlation: 0.241

Correlation with degree of change in lifestyle: *p*= 0.015; correlation: 0.317

### CDR-SB

Correlation with lifestyle at 20 weeks: *p* = 0.043; correlation: 0.251

Correlation with degree of change in lifestyle: *p* = 0.081; correlation: 0.205

### CDR-Global

Correlation with lifestyle at 20 weeks: *p* = 0.065; correlation: 0.221

Correlation with degree of change in lifestyle: *p* = 0.024; correlation: 0.286

### CGIC

Correlation with lifestyle at 20 weeks: *p* = 0.002

Correlation with degree of change in lifestyle: *p*= 0.0005

(CGIC tests are non-parametric analyses, so standard effect size calculations are not included for this measure.)

Also, we also found a significant correlation between dietary total fat intake and changes in the CGIC measure (*p* = 0.001), but this was not significant for the other three measures.

### Correlation of lifestyle index and biomarker data

In the current clinical trial, despite the inherent limitations of self-reported data, we found statistically significant correlations between the degree of lifestyle change (from baseline to 20 weeks) and the degree of change in many of the key biomarkers, as well as correlations between the degree of lifestyle change at 20 weeks and the degree of change in these biomarkers:

### Plasma Aβ42/40 ratio

Correlation with lifestyle at 20 weeks: *p* = 0.035; correlation: 0.306

Correlation with degree of change in lifestyle: *p* = 0.068; correlation: 0.266

### Glyc-A

Correlation with lifestyle at 20 weeks: *p* = 0.011; correlation: 0.363

Correlation with degree of change in lifestyle: *p* = 0.007; correlation: 0.383

### LDL-cholesterol

Correlation with lifestyle at 20 weeks: *p* < 0.0001; correlation: 0.678

Correlation with degree of change in lifestyle: *p* < 0.0001; correlation: 0.628

### Beta-Hydroxybutyrate (ketones)

Correlation with lifestyle at 20 weeks: *p* = 0.013; correlation: 0.372

Correlation with degree of change in lifestyle: *p* = 0.034; correlation: 0.320

### pTau 181

Correlation with lifestyle at 20 weeks: *p* = 0.228; correlation: 0.177

Correlation with degree of change in lifestyle: *p* = 0.135; correlation: 0.219

### GFAP/glial fibrillary acidic protein

Correlation with lifestyle at 20 weeks: *p* = 0.096; correlation: 0.243

Correlation with degree of change in lifestyle: *p* =0.351; correlation: 0.138

### What degree of lifestyle change is correlated with improvement in cognitive function tests?

What degree of lifestyle is needed to stop or improve the worsening of MCI or early dementia due to AD? In other words, what % of adherence to the lifestyle intervention was correlated with no change in MCI or dementia across both groups? Higher adherence than this degree of lifestyle change was associated with improvement in MCI or dementia.

#### ADAS-Cog

Correlation with lifestyle at 20 weeks: 71.4% adherence

#### CDR-SB

Correlation with lifestyle at 20 weeks: 120.6% adherence

#### CDR-Global:

Correlation with lifestyle at 20 weeks: 95.6%

### Microbiome results

There was a significant and beneficial change in the microbiome configuration in the intervention group but not in the control group.

Several taxa (groups of microorganisms) that increased only in the intervention group were consistent with those involved in reduced AD risk in other studies. For example, *Blautia,* which increased during the intervention in the intervention group, has previously been associated with a lower risk of AD, potentially due to its involvement in increasing γ-aminobutyric acid (GABA) production [37]. *Eubacterium* also increased during the intervention in the intervention group, and prior studies have identified *Eubacterium* genera (namely *Eubacterium fissicatena)* as a protective factor in AD [38].

Also, there was a decrease in relative abundance of taxa involved in increased AD risk in the intervention group, e.g., *Prevotella* and *Turicibacter*, the latter of which has been associated with relevant biological processes such as 5-HT production. *Prevotella* and *Turicibacter* have previously been shown to increase with disease progression, [39] and these taxa decreased over the course of the intervention.

These results support the hypothesis that the lifestyle intervention may beneficially modify specific microbial groups in the microbiome: increasing those that lower the risk of AD and decreasing those that increase the risk of AD. (Please see Supplement for more detailed information.)

## Discussion

We report the first randomized, controlled trial showing that an intensive multimodal lifestyle intervention may significantly improve cognition and function and may allay biological features in many patients with MCI or early dementia due to AD after 20 weeks.

After 20 weeks of a multimodal intensive lifestyle intervention, results of the primary analysis when all patients were included showed overall statistically significant differences between the intervention group and the randomized control group in cognition and function as measured by the CGIC (*p*= 0.001), CDR-SB (*p*= 0.032), and CDR Global (*p*= 0.037) tests and of borderline significance in the ADAS-Cog test (*p*= 0.053).

Three of these measures (CGIC, CDR Global, ADAS-Cog) showed improvement in cognition and function in the intervention group and worsening in the randomized control group, and one test (CDR-SB) showed less progression in the intervention group when compared to the control group which worsened in all four of these measures.

These differences were even clearer in a secondary sensitivity analysis when a mathematical outlier was excluded. These results showed statistically significant differences between groups in all four of these measures of cognition and function. Three of these measures showed improvement in cognition and function and one (CDR-SB) showed less deterioration when compared to the randomized control group, which worsened in all four of these measures.

The validity of these changes in cognition and function and possible biological mechanisms of improvement is supported by the observed changes in several clinically relevant biomarkers that showed statistically significant differences in a beneficial direction after 20 weeks when compared to the randomized control group.

One of the most clinically relevant biomarkers is the plasma Aβ42/40 ratio, which increased by 6.4% in the intervention group and decreased by 8.3% in the randomized control group after 20 weeks, and these differences were statistically significant (*p*= 0.003, two-tailed).

In the lecanemab trial, plasma levels of the Aβ42/40 biomarker increased in the intervention group over 18 months with the presumption that this reflected amyloid moving from the brain to the plasma [40]. We found similar results in the direction of change in the plasma Aβ42/40 ratio from this lifestyle intervention but in only 20 weeks. Conversely, this biomarker decreased in the control group (as in the lecanemab trial), which may indicate increased cerebral uptake of amyloid.

Other clinically relevant biomarkers also showed statistically significant differences (two-tailed) in a beneficial direction after 20 weeks when compared to the randomized control group. These include hemoglobin A1c, insulin, glycoprotein acetyls (GlycA), LDL-cholesterol, and β-Hydroxybutyrate (ketone bodies).

Improvement in these biomarkers provides more biological plausibility for the observed improvements in cognition and function as well as more insight into the possible mechanisms of improvement. This information may also help in predicting which patients are more likely to show improvements in cognition and function by making these intensive lifestyle changes.

Other relevant biomarkers were in a beneficial direction of change in the intervention group compared with the randomized control group after 20 weeks. These include pTau181, GFAP, CRP, SAA, and C-peptide. Telomere length increased in the intervention group and was essentially unchanged in the control group. These differences were not statistically significant even when there was an order of magnitude difference between groups (as with GFAP and pTau181) or an almost four-fold difference (as with CRP), but these changes were in a beneficial direction. At least in part, these findings may be due to a relatively small sample size and/or a short duration of only 20 weeks.

We found a statistically significant dose-response correlation between the degree of lifestyle changes in both groups (“lifestyle index”) and the degree of change in many of these biomarkers. This correlation was found in both the degree of change in lifestyle from baseline to 20 weeks as well as the lifestyle measured at 20 weeks. These correlations also add to the biological plausibility of these findings.

We also found a statistically significant dose-response correlation between the degree of lifestyle changes in both groups (“lifestyle index”) and changes in most measures of cognition and function testing. In short, the more these AD patients changed their lifestyle in the prescribed ways, the greater was the beneficial impact on their cognition and function. These correlations also add to the biological plausibility of these findings. This variation in adherence helps to explain in part why some patients in the intervention group improved and others did not, but there are likely other mechanisms that we do not fully understand that may play a role. These statistically significant correlations are especially meaningful given the greater variability of self-reported data, the relatively small sample size, and the short duration of the intervention.

These findings are consistent with earlier clinical trials in which we used this same lifestyle intervention and the same measure of lifestyle index and found significant dose-response correlations between this lifestyle index (i.e., the degree of lifestyle changes) and changes in the degree of coronary atherosclerosis (percent diameter stenosis) in coronary heart disease; [41, 45] changes in PSA levels and LNCaP cell growth in men with prostate cancer; [42] and changes in telomere length [43].

We also found significant differences between the intervention and control groups in several taxa (groups of micro-organisms) in the microbiome which may be beneficial.

There were no significant differences in depression scores as measured by PHQ-9 between the intervention and control groups. Therefore, reduction in depression is unlikely to account for the overall improvements in cognition and function seen in the intervention group patients.

We also found that substantial lifestyle changes were required to stop the progression of MCI in these patients. In the primary analysis, this ranged from 71.4% adherence for ADAS-Cog to 95.6% adherence for CDR-Global to 120.6% adherence for CDR-SB. In other words, extensive lifestyle changes were required to stop or improve cognition and function in these patients. This helps to explain why other studies of less-intensive lifestyle interventions may not have been sufficient to stop deterioration or improve cognition and function.

For example, comparing these results to those of the MIND-AD clinical trial provides more biological plausibility for both studies [44]. That is, more moderate multimodal lifestyle changes may slow the rate of worsening of cognition and function in MCI or early dementia due to early-stage AD, whereas more intensive multimodal lifestyle changes may result in overall average improvements in many measures of cognition and function when compared to a randomized usual-care control group in both clinical trials.

Lifestyle changes may provide additional benefits to patients on drug therapy. Anti-amyloid antibodies have shown modest effects on slowing progression, but they are expensive, have potential for adverse events, are not yet widely available, and do not result in overall cognitive improvement [40]. Perhaps there may be synergy from doing both.

## Limitations

This study has several limitations. Only 51 patients were enrolled and randomized in our study, and two of these patients (both in the intervention group) withdrew during the trial. Showing statistically significant differences across different tests of cognition and function and other measures despite the relatively small sample size suggests that the lifestyle intervention may be especially effective and has strong internal validity.

However, the smaller sample size limits generalizability, especially since there was much less racial and ethnic diversity in this sample than we strived to achieve. Also, we measured these differences despite the relative insensitivity of these measures, which might have increased the likelihood of a type II error.

Raters were blinded to the group assignment of the participants. However, unlike a double-blind placebo-controlled drug trial, it is not possible to blind subjects in a lifestyle intervention about whether or not they are receiving the intervention. This might have affected outcome measures, although to reduce positive expectations and because it was true, patients were told during the study that we did not know whether or not this lifestyle intervention would be beneficial, and we said that whatever we showed would be useful.

Also, 20 weeks is a relatively short time for any intervention with MCI or early dementia due to AD. We did not include direct measures of brain structure in this trial, so we cannot determine whether there were direct impacts on markers of brain pathology relevant to AD. However, surrogate markers such as the plasma Aβ42/40 ratio are becoming more widely accepted.

Not all patients in the intervention group improved. Of the 24 patients in the intervention group, 10 showed improvement as measured by the CGIC test, 7 were unchanged, and 7 worsened. In the control group, none improved, 8 were unchanged, and 17 worsened. In part, this may be explained by variations in adherence to the lifestyle intervention, as there was a significant relationship between the degree of lifestyle change and the degree of change in cognition and function across both groups. We hope that further research may further clarify other factors and mechanisms to help explain why cognition and function improved in some patients but not in others.

The findings on the degree of lifestyle change required to stop the worsening or improve cognition and function need to be interpreted with caution. Since data from both groups were combined, it was no longer a randomized trial for this specific analysis, so there could be unknown confounding influences. Also, it is possible that those with improved changes in cognition were better able to adhere to the intervention and thus have higher lifestyle indices.

## Conclusions

In summary, in persons with mild cognitive impairment or early dementia due to Alzheimer’s disease, comprehensive lifestyle changes may improve cognition and function in several standard measures after 20 weeks. In contrast, patients in the randomized control group showed overall worsening in all four measures of cognition and function during this time.

The validity of these findings was supported by the observed changes in plasma biomarkers and microbiome; the dose-response correlation of the degree of lifestyle change with the degree of improvement in all four measures of cognition and function; and the correlation between the degree of lifestyle change and the degree of changes in the Aβ42/40 ratio and the changes in some other relevant biomarkers in a beneficial direction.

Our findings also have implications for helping to prevent AD. Newer technologies, some aided by artificial intelligence, enable the probable diagnosis of AD years before it becomes clinically apparent. However, many people do not want to know if they are likely to get AD if they do not believe they can do anything about it. If intensive lifestyle changes may cause improvement in cognition and function in MCI or early dementia due to AD, then it is reasonable to think that these lifestyle changes may also help to prevent MCI or early dementia due to AD. Also, it may take less-extensive lifestyle changes to help prevent AD than to treat it. Other studies cited earlier on the effects of these lifestyle changes on diseases such as coronary heart disease support this conclusion. Clearly, intensive lifestyle changes rather than moderate ones seem to be required to improve cognition and function in those suffering from early-stage AD.

These findings support longer follow-up and larger clinical trials to determine the longer-term outcomes of this intensive lifestyle medicine intervention in larger groups of more diverse AD populations; why some patients beneficially respond to a lifestyle intervention better than others besides differences in adherence; as well as the potential synergy of these lifestyle changes and some drug therapies.

### Supplementary Information

Supplementary Material 1. 

## Data Availability

The datasets used and/or analyzed during the current study may be available from the corresponding author on reasonable request. Requesters will be asked to submit a study protocol, including the research question, planned analysis, and data required. The authors will evaluate this plan (i.e., relevance of the research question, suitability of the data, quality of the proposed analysis, planned or ongoing analysis, and other matters) on a case-by-case basis.

## References

[1] Livingston G, Huntley J, Sommerlad A, Ames D, Ballard C, Banerjee S, Brayne C, Burns A, Cohen-Mansfield J, Cooper C, Costafreda SG, Dias A, Fox N, Gitlin LN, Howard R, Kales HC, Kivimäki M, Larson EB, Ogunniyi A, Orgeta V, Ritchie K, Rockwood K, Sampson EL, Samus Q, Schneider LS, Selbæk G, Teri L, Mukadam N (2020). Dementia prevention, intervention, and care: 2020 report of the Lancet Commission. Lancet.

[2] Ornish D, Ornish A (2019). UnDo It.

[3] Dhana K, Agarwal P, James BD, Leurgans SE, Rajan KB, Aggarwal NT, Barnes LL, Bennett DA, Schneider JA. Healthy Lifestyle and Cognition in Older Adults With Common Neuropathologies of Dementia. JAMA Neurol. 2024. 10.1001/jamaneurol.2023.5491. Epub ahead of print. PMID: 38315471. 10.1001/jamaneurol.2023.5491 PMC10845037 38315471

[4] Morris MC, Evans DA, Tangney CC, Bienias JL, Wilson RS (2006). Associations of vegetable and fruit consumption with age-related cognitive change. Neurology.

[5] Morris MC, Evans DA, Bienias JL, Tangney CC, Bennett DA, Aggarwal N, Schneider J, Wilson RS (2003). Dietary fats and the risk of incident Alzheimer disease. Arch Neurol.

[6] Yu JT, Xu W, Tan CC, Andrieu S, Suckling J, Evangelou E, Pan A, Zhang C, Jia J, Feng L, Kua EH, Wang YJ, Wang HF, Tan MS, Li JQ, Hou XH, Wan Y, Tan L, Mok V, Tan L, Dong Q, Touchon J, Gauthier S, Aisen PS, Vellas B (2020). Evidence-based prevention of Alzheimer's disease: systematic review and meta-analysis of 243 observational prospective studies and 153 randomised controlled trials. J Neurol Neurosurg Psychiatry.

[7] Blumenthal JA, Smith PJ, Mabe S, Hinderliter A, Lin PH, Liao L (2019). Lifestyle and neurocognition in older adults with cognitive impairments: A randomized trial. Neurology.

[8] Ngandu T, Lehtisalo J, Solomon A, Levälahti E, Ahtiluoto S, Antikainen R, Bäckman L, Hänninen T, Jula A, Laatikainen T, Lindström J, Mangialasche F, Paajanen T, Pajala S, Peltonen M, Rauramaa R, Stigsdotter-Neely A, Strandberg T, Tuomilehto J, Soininen H, Kivipelto M (2015). A 2 year multidomain intervention of diet, exercise, cognitive training, and vascular risk monitoring versus control to prevent cognitive decline in at-risk elderly people (FINGER): a randomised controlled trial. Lancet.

[9] Rosenberg A, Ngandu T, Rusanen M, Antikainen R, Backman L, Havulinna S (2018). Multidomain lifestyle intervention benefits a large elderly population at risk for cognitive decline and dementia regardless of baseline characteristics: The FINGER trial. Alzheimers Dement.

[10] Solomon A, Turunen H, Ngandu T, Peltonen M, Levalahti E, Helisalmi S (2018). Effect of the apolipoprotein e genotype on cognitive change during a multidomain lifestyle intervention: a subgroup analysis of a randomized clinical trial. JAMA Neurol.

[11] Lehtisalo J, Rusanen M, Solomon A, Antikainen R, Laatikainen T, Peltonen M, et al. Effect of a multi-domain lifestyle intervention on cardiovascular risk in older people: the FINGER trial. Eur Heart J. 2022. 10.1093/eurheartj/ehab922. Epub 2022/01/21. PubMed PMID: 35051281. 10.1093/eurheartj/ehab922 PMC9156384 35051281

[12] Kivipelto M, Mangialasche F, Snyder HM, Allegri R, Andrieu S, Arai H (2020). World-Wide FINGERS Network: a global approach to risk reduction and prevention of dementia. Alzheimers Dement.

[13] Kivipelto M, Mangialasche F, Snyder H M, Allegri R, Andrieu S, Arai H, Baker L, Belleville S, Brodaty H, Brucki SM, Calandri I, Caramelli P, Chen C, Chertkow H, Chew E, Choi S H, Chowdhary N, Crivelli L, De La Torre R, Du Y, Dua T, Espeland M, Feldman H H, Hartmanis M, Hartmann T, Heffernan M, Henry C J, Hong C H, Håkansson K, Iwatsubo T, Jeong J H, Jimenez‐Maggiora G, Koo E H, Launer L J, Lehtisalo J, Lopera F, Martínez‐Lage P, Martins R, Middleton L, Molinuevo J L, Montero‐Odasso M, Moon S Y, Morales‐Pérez K, Nitrini R, Nygaard H B, Park Y K, Peltonen M, Qiu C, Quiroz Y T, Raman R, Rao N, Ravindranath V, Rosenberg A, Sakurai T, Salinas R M, Scheltens P, Sevlever G, Soininen H, Sosa A L, Suemoto C K, Tainta‐Cuezva M, Velilla L, Wang Y, Whitmer R, Xu X, Bain L J, Solomon A, Ngandu T, Carillo, M C. World‐Wide FINGERS Network: A global approach to risk reduction and prevention of dementia. Alzheimer's Dement. 2020, 10.1002/alz.12123.

[14] Yaffe K, Vittinghoff E, Dublin S, Peltz CB, Fleckenstein LE, Rosenberg DE, Barnes DE, Balderson BH, Larson EB. Effect of personalized risk-reduction strategies on cognition and dementia risk profile among older adults: the SMARRT randomized clinical trial. JAMA Intern Med. 2023:e236279. 10.1001/jamainternmed.2023.6279. Epub ahead of print. PMID: 38010725; PMCID: PMC10682943 10.1001/jamainternmed.2023.6279 PMC10682943 38010725

[15] Ornish D, Scherwitz LW, Billings JH, Brown SE, Gould KL, Merritt TA, Sparler S, Armstrong WT, Ports TA, Kirkeeide RL, Hogeboom C, Brand RJ (1998). Intensive lifestyle changes for reversal of coronary heart disease. JAMA.

[16] Ornish D, Scherwitz LW, Doody RS, Kesten D, McLanahan SM, Brown SE, DePuey E, Sonnemaker R, Haynes C, Lester J, McAllister GK, Hall RJ, Burdine JA, Gotto AM (1983). Effects of stress management training and dietary changes in treating ischemic heart disease. JAMA.

[17] Gould KL, Ornish D, Scherwitz L, Brown S, Edens RP, Hess MJ, Mullani N, Bolomey L, Dobbs F, Armstrong WT (1995). Changes in myocardial perfusion abnormalities by positron emission tomography after long-term, intense risk factor modification. JAMA.

[18] Dhana K, Evans DA, Rajan KB, Bennett DA, Morris MC (2020). Healthy lifestyle and the risk of Alzheimer dementia: Findings from 2 longitudinal studies. Neurology.

[19] McKhann GM, Knopman DS, Chertkow H, Hyman BT, Jack CR, Kawas CH, Klunk WE, Koroshetz WJ, Manly JJ, Mayeux R, Mohs RC, Morris JC, Rossor MN, Scheltens P, Carrillo MC, Thies B, Weintraub S, Phelps CH (2011). The diagnosis of dementia due to Alzheimer's disease: recommendations from the National Institute on Aging-Alzheimer's Association workgroups on diagnostic guidelines for Alzheimer's disease. Alzheimers Dement..

[20] Albert MS, DeKosky ST, Dickson D, Dubois B, Feldman HH, Fox NC (2011). The diagnosis of mild cognitive impairment due to Alzheimer’s disease: recommendations from the National Institute on Aging-Alzheimer’s Association workgroups on diagnostic guidelines for Alzheimer’s disease. Alzheimers Dement..

[21] McDonald K, Seltzer E, Lu M, Gaisenband SD, Fletcher C, McLeroth P, Saini KS (2023). Quantifying the impact of the COVID-19 pandemic on clinical trial screening rates over time in 37 countries. Trials.

[22] Tang HY, Vitiello MV, Perlis M, Mao JJ, Riegel B (2014). A pilot study of audio-visual stimulation as a self-care treatment for insomnia in adults with insomnia and chronic pain. Appl Psychophysiol Biofeedback.

[23] Horsley K (2016). Unlimited Memory.

[24] Morris MC, Evans DA, Bienias JL, Tangney CC, Bennett DA, Wilson RS (2003). Consumption of fish and n-3 fatty acids and risk of incident Alzheimer disease. Arch Neurol.

[25] Voulgaropoulou SD, van Amelsvoort T, Prickaerts J, Vingerhoets C (2019). The effect of curcumin on cognition in Alzheimer's disease and healthy aging: A systematic review of pre-clinical and clinical studies. Brain Res.

[26] Ringman JM, Frautschy SA, Teng E, Begum AN, Bardens J, Beigi M, Gylys KH, Badmaev V, Heath DD, Apostolova LG, Porter V, Vanek Z, Marshall GA, Hellemann G, Sugar C, Masterman DL, Montine TJ, Cummings JL, Cole GM (2012). Oral curcumin for Alzheimer's disease: tolerability and efficacy in a 24-week randomized, double blind, placebo-controlled study. Alzheimers Res Ther.

[27] Shea TB, Remington R (2015). Nutritional supplementation for Alzheimer's disease?. Curr Opin Psychiatry.

[28] Pradhan N, Singh C, Singh A (2021). Coenzyme Q10 a mitochondrial restorer for various brain disorders. Naunyn Schmiedebergs Arch Pharmacol.

[29] Harrison FE (2012). A critical review of vitamin C for the prevention of age-related cognitive decline and Alzheimer's disease. J Alzheimers Dis.

[30] Lauer AA, Grimm HS, Apel B, Golobrodska N, Kruse L, Ratanski E, et al. Mechanistic Link between Vitamin B12 and Alzheimer's Disease. Biomolecules. 2022;12(1). 10.3390/biom12010129. Epub 2022/01/22. PubMed PMID: 35053277; PubMed Central PMCID: PMCPMC8774227. 10.3390/biom12010129 PMC8774227 35053277

[31] Du K, Zheng X, Ma ZT, Lv JY, Jiang WJ, Liu MY (2021). Association of Circulating Magnesium Levels in Patients With Alzheimer's Disease From 1991 to 2021: A Systematic Review and Meta-Analysis. Front Aging Neurosci.

[32] Saitsu Y, Nishide A, Kikushima K, Shimizu K, Ohnuki K (2019). Improvement of cognitive functions by oral intake of Hericium erinaceus. Biomed Res.

[33] Mori K, Inatomi S, Ouchi K, Azumi Y, Tuchida T (2009). Improving effects of the mushroom Yamabushitake (Hericium erinaceus) on mild cognitive impairment: a double-blind placebo-controlled clinical trial. Phytother Res.

[34] Xiang S, Ji JL, Li S, Cao XP, Xu W, Tan L (2022). Efficacy and Safety of probiotics for the treatment of alzheimer's disease, mild cognitive impairment, and Parkinson's Disease: a systematic review and meta-analysis. Front Aging Neurosci.

[35] Fogelman I, West T, Braunstein JB, Verghese PB, Kirmess KM, Meyer MR, Contois JH, Shobin E, Ferber KL, Gagnon J, Rubel CE, Graham D, Bateman RJ, Holtzman DM, Huang S, Yu J, Yang S, Yarasheski KE (2023). Independent study demonstrates amyloid probability score accurately indicates amyloid pathology. Ann Clin Transl Neurol..

[36] Exploratory data analysis. John W. Tukey, 1977. Addison-Wesley, Reading MA. 10.1002/bimj.4710230408.

[37] Zhuang Z, Yang R, Wang W, Qi L, Huang T (2020). Associations between gut microbiota and Alzheimer's disease, major depressive disorder, and schizophrenia. J Neuroinflammation.

[38] Cammann D, Lu Y, Cummings MJ, Zhang ML, Cue JM, Do J, Ebersole J, Chen X, Oh EC, Cummings JL, Chen J (2023). Genetic correlations between Alzheimer's disease and gut microbiome genera. Sci Rep.

[39] Borsom EM, Conn K, Keefe CR, Herman C, Orsini GM, Hirsch AH, Palma Avila M, Testo G, Jaramillo SA, Bolyen E, Lee K, Caporaso JG, Cope EK (2023). Predicting Neurodegenerative Disease Using Prepathology Gut Microbiota Composition: a Longitudinal Study in Mice Modeling Alzheimer's Disease Pathologies. Microbiol Spectr.

[40] van Dyck CH, Swanson CJ, Aisen P, Bateman RJ, Chen C, Gee M, Kanekiyo M, Li D, Reyderman L, Cohen S, Froelich L, Katayama S, Sabbagh M, Vellas B, Watson D, Dhadda S, Irizarry M, Kramer LD, Iwatsubo T (2023). Lecanemab in Early Alzheimer's Disease. N Engl J Med.

[41] Ornish D, Scherwitz LW, Billings JH, Brown SE, Gould KL, Merritt TA, Sparler S, Armstrong WT, Ports TA, Kirkeeide RL, Hogeboom C, Brand RJ (1998). Intensive lifestyle changes for reversal of coronary heart disease. JAMA.

[42] Ornish D, Weidner G, Fair WR, Marlin R, Pettengill EB, Raisin CJ, Dunn-Emke S, Crutchfield L, Jacobs FN, Barnard RJ, Aronson WJ, McCormac P, McKnight DJ, Fein JD, Dnistrian AM, Weinstein J, Ngo TH, Mendell NR, Carroll PR (2005). Intensive lifestyle changes may affect the progression of prostate cancer. J Urol..

[43] Ornish D, Lin J, Chan JM, Epel E, Kemp C, Weidner G, Marlin R, Frenda SJ, Magbanua MJM, Daubenmier J, Estay I, Hills NK, Chainani-Wu N, Carroll PR, Blackburn EH (2013). Effect of comprehensive lifestyle changes on telomerase activity and telomere length in men with biopsy-proven low-risk prostate cancer: 5-year follow-up of a descriptive pilot study. Lancet Oncol.

[44] Kivipelto M et al. Multimodal preventive trial for Alzheimer’s disease. Alzheimer’s Dement. 2021;17(Suppl.10):e056105. https://alz-journals.onlinelibrary.wiley.com/doi/abs/10.1002/alz.056105.

[45] Ornish D, Brown SE, Scherwitz LW, Billings JH, Armstrong WT, Ports TA, McLanahan SM, Kirkeeide RL, Brand RJ, Gould KL (1990). Can lifestyle changes reverse coronary heart disease? The Lifestyle Heart Trial. Lancet.

