# Supplementary material for: Effects of intensive lifestyle changes on the progression of mild cognitive impairment or early dementia due to Alzheimer’s disease: a randomized, controlled clinical trial
Source: Alzheimers Res Ther. 2024 Jun 7;16:122. doi: 10.1186/s13195-024-01482-z (PMC11157928; doi:10.1186/s13195-024-01482-z)
Supplement: Supplementary file 1 — Supplementary Material 1. [file 13195_2024_1482_MOESM1_ESM.docx]

**Supplemental Materials**

**Methods of Secondary Outcomes Measures:**

Behavior

Depression scores were assessed using the Patient Health Questionnaire (PHQ-9). A 3-day diet diary and a lifestyle adherence survey were also completed.

Blood-Based Biomarkers:

We measured selected biomarkers that play a role in the pathophysiology of AD, as further described in the Results section. The plasma Aβ42/40 ratio, phosphorylated tau181 (pTau181), GFAP/Glial Fibrillary Acidic Protein, hemoglobin A1c, insulin, C-reactive protein (CRP), Serum Amyloid A/SAA, and C‑Peptide were assessed at the Arnold and Tanzi Laboratories (Harvard Medical School/Massachusetts General Hospital). Telomere length was measured at the Elizabeth Blackburn Laboratory (UCSF).^[[1]](#endnote-1)^ We used quantitative PCR to measure average telomere length, represented by the ratio of telomere signal vs. a single copy gene signal (T/S). LDL‑cholesterol, glycoprotein acetyls (GlycA), and beta-hydroxybutyrate were measured at the Kaddurah-Daouk-led “Alzheimer Disease Metabolomics Consortium” and the Alzheimer Gut Microbiome Project (Duke Medical Center).^[[2]](#endnote-2)^ ^[[3]](#endnote-3)^ All those doing these assessments were blind to participant identity or treatment group assignment.

An increase in the plasma Aβ42/40 ratio level is considered to be beneficial as an indication that amyloid is moving from the brain to the plasma. ^[[4]](#endnote-4)^ Higher hemoglobin A1c levels are significantly associated with an increased risk of AD.^[[5]](#endnote-5)^ Insulin resistance promotes the development of cognitive dysfunction by hyperinsulinemia and impaired insulin signaling. Elevated levels of C-peptide are linked with insulin resistance and an increased risk of AD.^[[6]](#endnote-6)^ β‑Hydroxybutyrate (βOHB), the most prevalent type of ketone in the human body, may be involved in the pathogenesis of [cognitive disorders](https://www.sciencedirect.com/topics/neuroscience/cognitive-disorders), especially AD, through a variety of protective mechanisms, such as enhancing [mitochondrial metabolism](https://www.sciencedirect.com/topics/neuroscience/mitochondrial-respiration), regulating signaling molecule, increasing [histone acetylation](https://www.sciencedirect.com/topics/neuroscience/histone-acetylation), affecting the metabolism of Aβ and tau proteins, inhibiting inflammation and lipid metabolism, and regulating [intestinal microbes](https://www.sciencedirect.com/topics/pharmacology-toxicology-and-pharmaceutical-science/intestine-flora).^[[7]](#endnote-7)^ APOE is a strong risk factor for AD (AD) and is associated with higher low-density lipoprotein cholesterol (LDL-C) levels. LDL-C is also associated with a higher risk of AD independent of APOE.^[[8]](#endnote-8)^ Plasma pTau181 is significantly elevated in people with AD compared to control individuals.^[[9]](#endnote-9)^ Plasma pTau181 was found to be increased in people who convert to AD dementia from MCI, compared with those who converted to dementia due to non‐AD diseases.  Higher levels of p-Tau 181 indicate an increased risk of progression of AD.^[[10]](#endnote-10)^ GFAP increases astrogliosis and neuroinflammation, thereby increasing the risk of progression of AD.^[[11]](#endnote-11)^ CRP is a marker of inflammation (lower indicates improvement). Elevation in CRP is associated with an increased risk of AD, especially in ApoE4 carriers.^[[12]](#endnote-12)^ ^[[13]](#endnote-13)^ SAA is associated with the severity of senile early cognitive impairment; a marker for inflammation and astrogliosis; lower = beneficial.^[[14]](#endnote-14)^ Glycoprotein acetyls (GlycA) is a composite biomarker that integrates the protein levels and glycosylation states of several of the most abundant acute phase proteins in serum. As such, this may allow for a more stable measure of inflammation with low intra-individual variability and may provide a more sensitive measure than CRP.^[[15]](#endnote-15)^ Shorter telomeres are associated with an increased risk of AD.^[[16]](#endnote-16)^

Blood samples were drawn after an overnight fast, plasma was prepared at each site following standardized protocols, frozen and stored at -80 degrees C, to allow measurement of biomarkers.^[[17]](#endnote-17)^ Whole blood collected in EDTA tubes was used for telomere length.^[[18]](#endnote-18)^ ^[[19]](#endnote-19)^ One patient in the randomized control group did not provide sufficient blood to analyze and was unwilling to be redrawn.

Microbiome Taxa (Organisms):

Subject gut microbiome specimens were collected under the American Gut Project.^[[20]](#endnote-20)^ Subjects were instructed to use a sterile Swube to collect fecal material from used toilet paper, and to smear the material on a sterile FOBT card to fix nucleic acid. Samples were extracted in triplicate, and the 16S V4 rRNA region was sequenced following the Earth Microbiome Project protocol^[[21]](#endnote-21)^ on the Illumina MiSeq platform, and deposited in Qiita under study 10317. Replicates were sequenced and processed with Deblur^[[22]](#endnote-22)^ separately, then merged. Amplicon Sequence Variants were filtered against Greengenes2 2022.10.^[[23]](#endnote-23)^ A rarefaction level of 1187 was selected which preserved all merged subject samples. Unweighted UniFrac was computed.^[[24]](#endnote-24)^ Procrustes analysis was performed using q2-diversity 2023.9.^[[25]](#endnote-25)^ Convex hulls were computed using q2-convexhull (unversioned). Mantel tests computed with scikit-bio version 0.5.9, unless otherwise mentioned.

**Patient Recruitment**

| Total Potential Subjects Screened | 1585 |
| --- | --- |
| Enrolled | 51 |
| Declined | 102 |
| Ineligible | 1300 |
| Incomplete screening when enrollment closed | 132 |

| Recruitment Source for Enrolled | 51 |
| --- | --- |
| Clinical trials database | 8 |
| Friend / word of mouth | 8 |
| Linea | 1 |
| Physician / medical provider | 30 |
| Podcast / YouTube / Radio / TV | 2 |
| Search engine | 1 |
| Website | 1 |

Statistical Methods

ADAS-Cog, CDR-SB, and CDR-Global scales are numeric, so the analyses compared the change scores from baseline to 20 weeks using a Mann-Whitney-Wilcoxon rank-sum test. ADAS-Cog and CDR-SB are continuous variables and the CDR-Global is ordinal. CGIC was recorded as a qualitative change over baseline, so Fisher’s exact test was used to compare the groups using the 20 week values. PHQ-9 scores were also analyzed using a Mann-Whitney-Wilcoxon rank-sum test. Stata Version 16.1 was used for all analyses other than microbiome analyses which used SciPy v1.10.

Since there is an increasingly large body of research showing that similar multimodal lifestyle changes such as exercise, healthy diet, stress management, and social support may reduce the risk of dementia and slow its progression, as well as beneficially affect many other chronic diseases, the direction of the results of these lifestyle changes on cognition and function testing is highly unlikely to be detrimental. In this context, the use of one-tailed statistical tests is appropriate and more likely to be clinically relevant.^[[26]](#endnote-26)^ For these reasons, and to reduce the risk of type 2 errors, we used one-tailed tests for describing changes in cognition and function. Changes in biomarkers were secondary outcomes presented to support the primary outcome results.  Therefore, we did not apply a multiple testing correction and only nominal p-values are reported.  Two-tailed tests were used to analyze changes in biomarkers since the direction of change in these measures as well as PHQ-9 testing *a priori* was unclear, as well as PHQ-9 testing.

Correlations with Degree of Lifestyle Changes:

To assess the correlation between the degree of lifestyle change and the degree of change in measures of cognition and function and also with biomarkers, we used a formula (please see Supplement) devised in earlier clinical trials to correlate the degree of all four modalities of these lifestyle changes (diet, exercise, stress management, and social support) with a variety of endpoint measures called a “lifestyle index.”^[[27]](#endnote-27)^ In these earlier studies, we found a statistically significant correlation between the degree of lifestyle index change and the degree of change in percent diameter stenosis of coronary atherosclerosis; of changes in PSA levels in men with prostate cancer; in changes in LNCaP cell growth; and in telomere length.^[[28]](#endnote-28)^ ^[[29]](#endnote-29)^

For the numeric variables (ADAS-cog, CDR-SB, and CDR Global and all the biomarkers as well as the Lifestyle Index at 20 weeks and the change in the Lifestyle Index from baseline to 20 weeks), the Spearman rank correlation was used. The CGIC is ordinal, so the non-parametric (rank-based) Jonckheere-Terpstra test for trend was used in the values of the change in biomarkers as well as the Lifestyle Index tests.

To determine the degree of lifestyle change needed to stop or improve worsening of cognition and function in these patients across both groups, we assessed the point at which 20-week values of the lifestyle index or change in the lifestyle index corresponded to no decline in cognitive performance. The numeric cognition and functioning scores (ADAS-cog, CDR Sum of Boxes and CDR Global) were regressed on both the lifestyle index at 20 weeks and the change in the lifestyle index from baseline to 20 weeks. The form of the linear regression equations for either lifestyle variable is: change in cognition = a + b*(lifestyle). Solving for the value of the lifestyle variable associated with no change in cognition gives: lifestyle=-a/b.

**Lifestyle/Adherence Formula:**

We calculated the mean percentage of adherence according to the following formula:


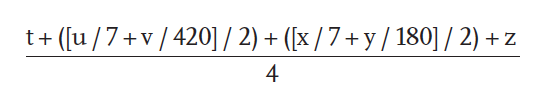


where t indicates support-group attendance (% sessions attended/month), u indicates stress-reduction activity days per week, v indicates stress-reduction activity minutes per week, x exercise days per week, y exercise minutes per week, and z adherence to diet. Adherence to exercise and stress management was assessed by dividing the self-reported number of minutes per week by the average number of minutes requested in the intervention per week (180 and 420, respectively). Dietary adherence was calculated in the following way:


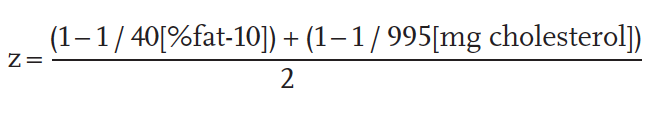


Increases in scores (mean percentage of adherence) reflect improved adherence to the recommended four components of the intervention—nutrition, stress management, exercise, and social support. We included dietary fat and cholesterol as surrogate measures of dietary adherence (not because they are the most important dietary components) because these can be quantified as continuous variables. We used a semiquantitative food frequency questionnaire to assess consumption of other recommended foods. A lifestyle index score of 1.0 suggested 100% adherence. Scores could exceed 1.0 for individuals who completed more hours of exercise and stress management than required.

**Additional Microbiome Analyses:**

Overall microbiome composition after 20 weeks changed significantly from baseline in the intervention but not the control group. These results suggest the intervention affects the gut microbiome, and further show that subjects undergoing intervention change in the same way and towards a more similar microbiome configuration than is exhibited at baseline. The specific observations that support this idea are as follows.

First, we observed that the intervention group exhibited a significantly greater pre-post difference in the whole microbiome over time (Mann Whitney U, u=30; p=1.1e-7) using leave-one-out Procrustes analysis (supplemental methods) (figure 5).


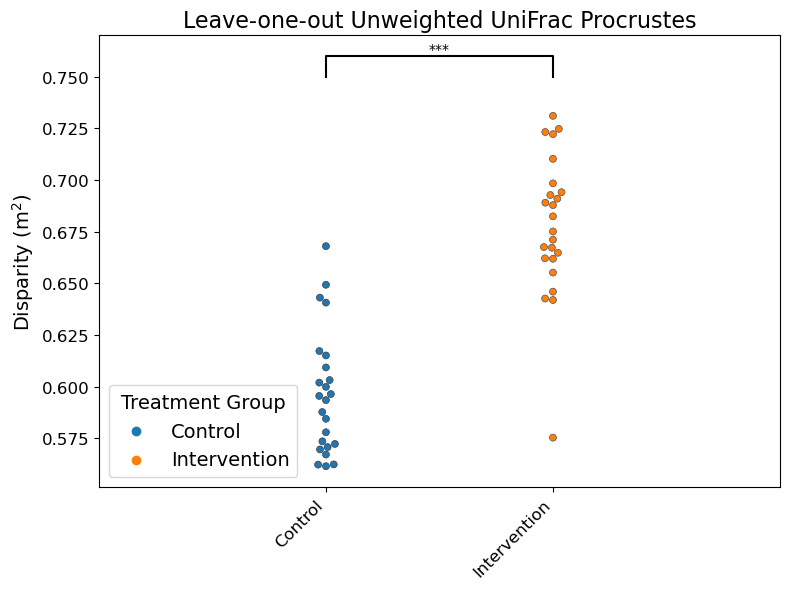


Figure 5: plot depicting the disparity distribution from running a leave-one-out Procrustes analysis on the unweighted UniFrac distance matrix, stratified by treatment group. This plot shows that the differences pre-post at the whole microbiome level are smaller in the control group than the intervention group, suggesting that the intervention has an effect at the whole microbiome level.

We further observed a significant reduction in the differences among subject microbiomes with the intervention (Mann Whitney U, p=3.06e-9) with a leave-one-out Convex Hull analysis (supplemental methods). No difference in the distribution of Convex Hull volumes was observed in the control group between baseline and 20 weeks (figure 6, Mann Whitney U, u=0; p=0.392). These results suggest that no change occurred in the control group, but the significant reduction in volume of microbiome configuration space occupied by the intervention subjects suggests they change to similar microbiomes overall during the intervention.


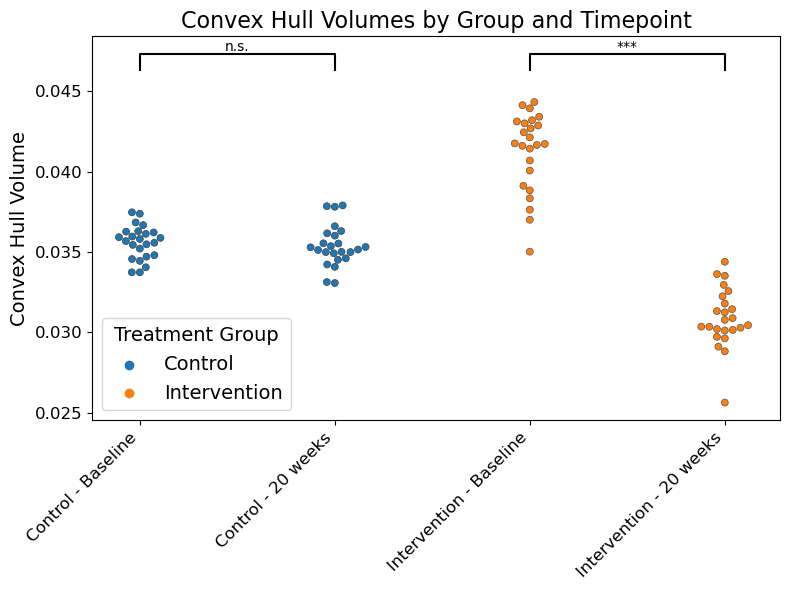


Figure 6: plot depicting total volumes from a leave-one-out convex hull analysis on principal coordinates of the unweighted UniFrac distance matrix, stratified by timepoint and treatment group. Significance levels are denoted by asterisks: '***' indicates a p-value < 0.0001, signifying a highly statistically significant correlation between groups based on the Mann-Whitney U test, while “n.s.” indicates no significant correlation between groups. This analysis demonstrates that the control group occupied the same volume of microbiome configuration space pre- and post-treatment, but that the treatment group started off with greater spread and converged on smaller spread after treatment, suggesting that the treatment resulted in more similar microbiomes.

Also, we adapted the BIRDMAn Bayesian inferential regression differential abundance model to identify the Amplicon Sequence Variants (ASV) that changed specifically between the pre-intervention timepoint and the post-intervention timepoint in the intervention but not the control group.^[[30]](#endnote-30)^ This sensitive statistical method designed for microbiome data tests which ASVs differ between timepoints. ASVs associating with both timepoints were detected (figure 7). Positive values are associated with baseline, and negative values associated with 20-weeks. A log ratio was formed from the differential taxa, which exhibits a significant difference between timepoints for the intervention group but not the control group (figure 8, paired t-test, Control t=-0.383, p=0.706; Intervention t=-5.88; p=1.17e-5).


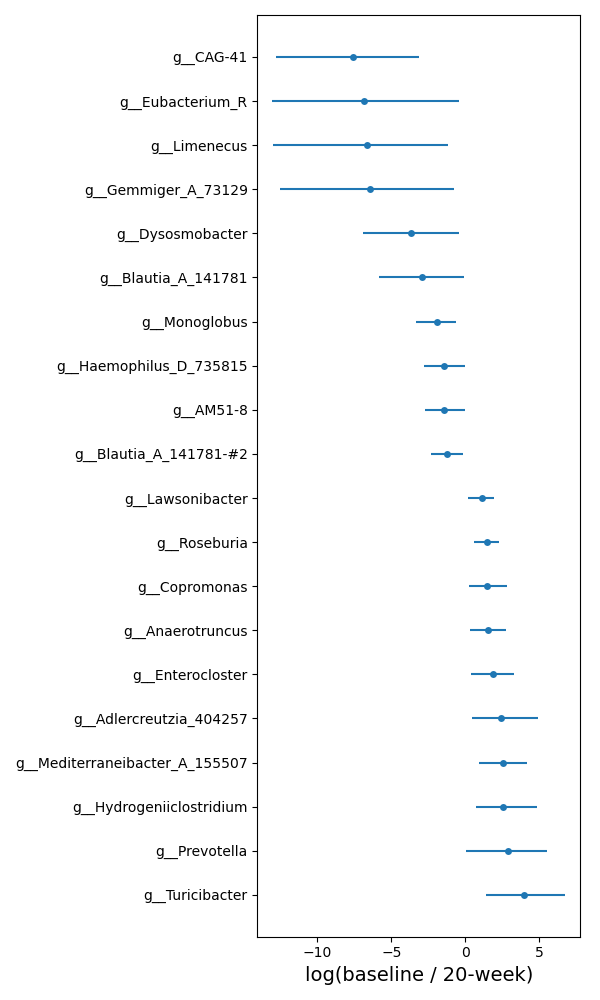


Figure 7: plot visualizing the top 10 and bottom 10 differentially abundant taxa identified using BIRDMAn within the intervention group with timepoint as a fixed effect and subject as a random effect.


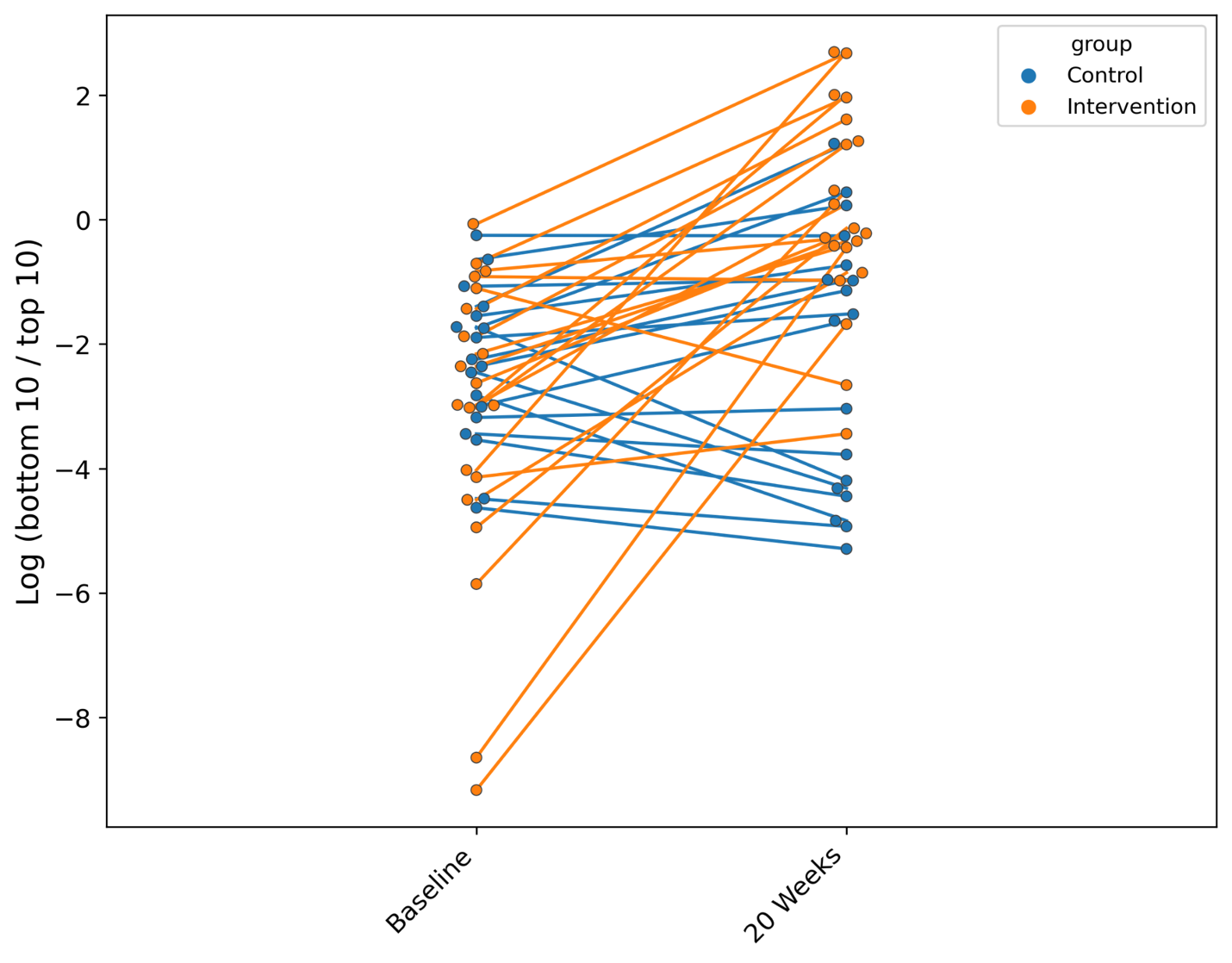


Figure 8: plot depicting log-ratios of differentially abundant taxa at baseline and 20 weeks using the ASVs identified as differential between timepoints in the intervention group. n=41 subjects at baseline, and n=43 subjects at 20 weeks had non-zero sums for the numerator and denominator taxa. To account for this, only subjects with log ratios at both timepoints were included (n=38). Significance was tested with a paired T-test which assumes related samples (Control: t=-0.383, p=0.706; Intervention t=-5.88, p=1.17e-5). These results suggest the intervention is associated with changes in the microbiome, which were not observed in the control group, and offers potential taxa of interest for further study.

In humans, there is mixed evidence for interpretation of *Prevotella* in AD patients, with some evidence for its enrichment in AD patients and negative correlation with cognition and function [10.3233/JAD-201040], and some evidence for its negative correlation with cognitive impairment,^[[31]](#endnote-31)^ suggesting that further investigation using higher-resolution techniques such as shotgun metagenomics would be rewarding. *Turicibacter* is implicated in regulation of gut serotonin (5-HT) production, also prompting further investigation.^[[32]](#endnote-32)^ Therefore, follow-up studies to determine whether these specific taxa or individual strains within them act as biomarkers that predict AD progression or response to treatments for AD would be valuable.

Taken together, these four complementary statistical approaches with different underlying bases suggest that the intervention changes the overall microbiome more than would be expected by chance over the same period in ways that may be beneficial, and the fourth also provides leads on specific taxa for follow-up.

As a complementary approach, we used Mantel tests to identify relationships between the control and intervention group at the whole microbiome level [PMID: 6018555]. The Mantel correlations between timepoints within groups were significant, with greater variation explained in the control group (Mantel using Pearson, control r^2^=0.312, p=0.001; intervention r^2^=0.243, p=0.001). This analysis demonstrates that the change in the microbiome is greater in the intervention group than in the control group, because the subject-subject relationships are less correlated in the intervention group.

The differences at the whole microbiome level within subject between pre- and post-intervention timepoints differ significantly between the control and intervention groups (Mann Whitney U, u=178722; p=0.023). The null hypothesis for this analysis is that differences in microbiomes between timepoints within an individual are not affected by group membership.

1. Lin J, Smith DL, Esteves K, Drury S. Telomere length measurement by qPCR - Summary of critical factors and recommendations for assay design. Psychoneuroendocrinology. 2019 Jan;99:271-278. doi: 10.1016/j.psyneuen.2018.10.005. Epub 2018 Oct 10. PMID: 30343983; PMCID: PMC6363640. [↑](#endnote-ref-1)
2. Soininen, P., Kangas, A. J., Würtz, P., Suna, T. & Ala-Korpela, M. Quantitative serum nuclear magnetic resonance metabolomics in cardiovascular epidemiology and genetics. Circ. Cardiovasc. Genet. 8, 192–206 (2015). [↑](#endnote-ref-2)
3. Soininen, P. et al. High-throughput serum NMR metabonomics for cost-effective holistic studies on systemic metabolism. Analyst 134, 1781–1785 (2009). [↑](#endnote-ref-3)
4. Doecke JD, Pérez-Grijalba V, Fandos N, Fowler C, Villemagne VL, Masters CL, Pesini P, Sarasa M; AIBL Research Group. Total Aβ_42_/Aβ_40_ ratio in plasma predicts amyloid-PET status, independent of clinical AD diagnosis. Neurology. 2020 Apr 14;94(15):e1580-e1591. doi: 10.1212/WNL.0000000000009240. Epub 2020 Mar 16. PMID: 32179698; PMCID: PMC7251518.Bottom of Form [↑](#endnote-ref-4)
5. Noguchi-Shinohara M, Yuki-Nozaki S, Abe C, Mori A, Horimoto M, Yokogawa M, Ishida N, Suga Y, Ishizaki J, Ishimiya M, Nakamura H, Komai K, Nakamura H, Shibata M, Ohara T, Hata J, Ninomiya T, Yamada M; Japan Prospective Studies Collaboration for Aging and Dementia (JPSC-AD) study group. Diabetes Mellitus, Elevated Hemoglobin A1c, and Glycated Albumin Are Associated with the Presence of All-Cause Dementia and Alzheimer's Disease: The JPSC-AD Study. J Alzheimers Dis. 2022;85(1):235-247. doi: 10.3233/JAD-215153. PMID: 34806607. [↑](#endnote-ref-5)
6. Arnold SE, Arvanitakis Z, Macauley-Rambach SL, Koenig AM, Wang HY, Ahima RS, Craft S, Gandy S, Buettner C, Stoeckel LE, Holtzman DM, Nathan DM. Brain insulin resistance in type 2 diabetes and Alzheimer disease: concepts and conundrums. Nat Rev Neurol. 2018;14(3):168-81. Epub 2018/01/30. doi: 10.1038/nrneurol.2017.185. PubMed PMID: 29377010; PMCID: PMC6098968. [↑](#endnote-ref-6)
7. Wang JH, Guo L, Wang S, Yu NW, Guo FQ. The potential pharmacological mechanisms of β-hydroxybutyrate for improving cognitive functions. Curr Opin Pharmacol. 2022 Feb;62:15-22. doi: 10.1016/j.coph.2021.10.005. Epub 2021 Dec 7. PMID: 34891124. [↑](#endnote-ref-7)
8. Wingo AP, Vattathil SM, Liu J, Fan W, Cutler DJ, Levey AI, Schneider JA, Bennett DA, Wingo TS. LDL cholesterol is associated with higher AD neuropathology burden independent of APOE. J Neurol Neurosurg Psychiatry. 2022 Jun 30;93(9):930–8. doi: 10.1136/jnnp-2021-328164. Epub ahead of print. PMID: 35772923; PMCID: PMC9380478. [↑](#endnote-ref-8)
9. Kivisäkk P, Carlyle BC, Sweeney T, Trombetta BA, LaCasse K, El-Mufti L, Tuncali I, Chibnik LB, Das S, Scherzer CR, Johnson KA, Dickerson BC, Gomez-Isla T, Blacker D, Oakley DH, Frosch MP, Hyman BT, Aghvanyan A, Bathala P, Campbell C, Sigal G, Stengelin M, Arnold SE. Plasma biomarkers for diagnosis of Alzheimer's disease and prediction of cognitive decline in individuals with mild cognitive impairment. Front Neurol. 2023 Mar 2;14:1069411. doi: 10.3389/fneur.2023.1069411. PMID: 36937522; PMCID: PMC10018178. [↑](#endnote-ref-9)
10. Meng J, Lei P. Plasma pTau181 as a biomarker for Alzheimer's disease. MedComm (2020). 2020 May 22;1(1):74-76. doi: 10.1002/mco2.1. PMID: 34766110; PMCID: PMC8489665. [↑](#endnote-ref-10)
11. Kim KY, Shin KY, Chang KA. GFAP as a Potential Biomarker for Alzheimer's Disease: A Systematic Review and Meta-Analysis. Cells. 2023 May 4;12(9):1309. doi: 10.3390/cells12091309. PMID: 37174709; PMCID: PMC10177296. [↑](#endnote-ref-11)
12. Royall DR. Reader Response: Impact of C-Reactive Protein on Cognition and Alzheimer Disease Biomarkers in Homozygous APOE ɛ4 Carriers. Neurology. 2022 Nov 15;99(20):918-919. doi: 10.1212/WNL.0000000000201508. PMID: 36376090. [↑](#endnote-ref-12)
13. Watanabe Y, Kitamura K, Nakamura K, Sanpei K, Wakasugi M, Yokoseki A, Onodera O, Ikeuchi T, Kuwano R, Momotsu T, Narita I, Endo N. Elevated C-Reactive Protein Is Associated with Cognitive Decline in Outpatients of a General Hospital: The Project in Sado for Total Health (PROST). Dement Geriatr Cogn Dis Extra. 2016 Jan 19;6(1):10-9. doi: 10.1159/000442585. PMID: 26933436; PMCID: PMC4772636. [↑](#endnote-ref-13)
14. Kindy MS, Yu J, Guo JT, Zhu H. Apolipoprotein Serum Amyloid A in Alzheimer's Disease. J Alzheimers Dis. 1999 Oct;1(3):155-167. doi: 10.3233/jad-1999-1303. PMID: 12214001. [↑](#endnote-ref-14)
15. Regan P, McClean PL, Smyth T, Doherty M. Early Stage Glycosylation Biomarkers in Alzheimer's Disease. Medicines (Basel). 2019 Sep 3;6(3):92. doi: 10.3390/medicines6030092. PMID: 31484367; PMCID: PMC6789538. [↑](#endnote-ref-15)
16. Rodríguez-Fernández B, Vilor-Tejedor N, Arenaza-Urquijo EM, Sánchez-Benavides G, Suárez-Calvet M, Operto G, Minguillón C, Fauria K, Kollmorgen G, Suridjan I, de Moura MC, Piñeyro D, Esteller M, Blennow K, Zetterberg H, De Vivo I, Molinuevo JL, Navarro A, Gispert JD, Sala-Vila A, Crous-Bou M; ALFA study. Genetically predicted telomere length and Alzheimer's disease endophenotypes: a Mendelian randomization study. Alzheimers Res Ther. 2022 Nov 7;14(1):167. doi: 10.1186/s13195-022-01101-9. PMID: 36345036; PMCID: PMC9641781. [↑](#endnote-ref-16)
17. Lin, J., et al., Analyses and comparisons of telomerase activity and telomere length in human T and B cells: insights for epidemiology of telomere maintenance. J Immunol Methods, 2010. **352**(1-2): p. 71-80. [↑](#endnote-ref-17)
18. Cawthon, R.M., Telomere measurement by quantitative PCR. Nucleic Acids Res, 2002. **30**(10): p. e47. [↑](#endnote-ref-18)
19. Details of the telomere length assay can be found at Telomere Research Network: <https://trn.tulane.edu/wp-content/uploads/sites/445/2021/07/Lin-qPCR-protocol-01072020.pdf> [↑](#endnote-ref-19)
20. McDonald D, Hyde E, Debelius JW, Morton JT, Gonzalez A, Ackermann G, Aksenov AA, Behsaz B, Brennan C, Chen Y, DeRight Goldasich L, Dorrestein PC, Dunn RR, Fahimipour AK, Gaffney J, Gilbert JA, Gogul G, Green JL, Hugenholtz P, Humphrey G, Huttenhower C, Jackson MA, Janssen S, Jeste DV, Jiang L, Kelley ST, Knights D, Kosciolek T, Ladau J, Leach J, Marotz C, Meleshko D, Melnik AV, Metcalf JL, Mohimani H, Montassier E, Navas-Molina J, Nguyen TT, Peddada S, Pevzner P, Pollard KS, Rahnavard G, Robbins-Pianka A, Sangwan N, Shorenstein J, Smarr L, Song SJ, Spector T, Swafford AD, Thackray VG, Thompson LR, Tripathi A, Vázquez-Baeza Y, Vrbanac A, Wischmeyer P, Wolfe E, Zhu Q; American Gut Consortium; Knight R. American Gut: an Open Platform for Citizen Science Microbiome Research. mSystems. 2018 May 15;3(3):e00031-18. doi: 10.1128/mSystems.00031-18. PMID: 29795809; PMCID: PMC5954204. [↑](#endnote-ref-20)
21. Caporaso JG, Lauber CL, Walters WA, Berg-Lyons D, Lozupone CA, Turnbaugh PJ, Fierer N, Knight R. Global patterns of 16S rRNA diversity at a depth of millions of sequences per sample. Proc Natl Acad Sci U S A. 2011 Mar 15;108 Suppl 1(Suppl 1):4516-22. doi: 10.1073/pnas.1000080107. Epub 2010 Jun 3. PMID: 20534432; PMCID: PMC3063599. [↑](#endnote-ref-21)
22. Amir A, McDonald D, Navas-Molina JA, Kopylova E, Morton JT, Zech Xu Z, Kightley EP, Thompson LR, Hyde ER, Gonzalez A, Knight R. Deblur Rapidly Resolves Single-Nucleotide Community Sequence Patterns. mSystems. 2017 Mar 7;2(2):e00191-16. doi: 10.1128/mSystems.00191-16. PMID: 28289731; PMCID: PMC5340863. [↑](#endnote-ref-22)
23. McDonald D, Jiang Y, Balaban M, Cantrell K, Zhu Q, Gonzalez A, Morton JT, Nicolaou G, Parks DH, Karst SM, Albertsen M, Hugenholtz P, DeSantis T, Song SJ, Bartko A, Havulinna AS, Jousilahti P, Cheng S, Inouye M, Niiranen T, Jain M, Salomaa V, Lahti L, Mirarab S, Knight R. Greengenes2 unifies microbial data in a single reference tree. Nat Biotechnol. 2023 Jul 27:10.1038/s41587-023-01845-1. doi: 10.1038/s41587-023-01845-1. Epub ahead of print. Erratum in: Nat Biotechnol. 2023 Oct 18;: PMID: 37500913; PMCID: PMC10818020. [↑](#endnote-ref-23)
24. Sfiligoi I, Armstrong G, Gonzalez A, McDonald D, Knight R. Optimizing UniFrac with OpenACC Yields Greater Than One Thousand Times Speed Increase. mSystems. 2022 Jun 28;7(3):e0002822. doi: 10.1128/msystems.00028-22. Epub 2022 May 31. PMID: 35638356; PMCID: PMC9239203. [↑](#endnote-ref-24)
25. Bolyen E, Rideout JR, Dillon MR, Bokulich NA, Abnet CC, Al-Ghalith GA, Alexander H, Alm EJ, Arumugam M, Asnicar F, Bai Y, Bisanz JE, Bittinger K, Brejnrod A, Brislawn CJ, Brown CT, Callahan BJ, Caraballo-Rodríguez AM, Chase J, Cope EK, Da Silva R, Diener C, Dorrestein PC, Douglas GM, Durall DM, Duvallet C, Edwardson CF, Ernst M, Estaki M, Fouquier J, Gauglitz JM, Gibbons SM, Gibson DL, Gonzalez A, Gorlick K, Guo J, Hillmann B, Holmes S, Holste H, Huttenhower C, Huttley GA, Janssen S, Jarmusch AK, Jiang L, Kaehler BD, Kang KB, Keefe CR, Keim P, Kelley ST, Knights D, Koester I, Kosciolek T, Kreps J, Langille MGI, Lee J, Ley R, Liu YX, Loftfield E, Lozupone C, Maher M, Marotz C, Martin BD, McDonald D, McIver LJ, Melnik AV, Metcalf JL, Morgan SC, Morton JT, Naimey AT, Navas-Molina JA, Nothias LF, Orchanian SB, Pearson T, Peoples SL, Petras D, Preuss ML, Pruesse E, Rasmussen LB, Rivers A, Robeson MS 2nd, Rosenthal P, Segata N, Shaffer M, Shiffer A, Sinha R, Song SJ, Spear JR, Swafford AD, Thompson LR, Torres PJ, Trinh P, Tripathi A, Turnbaugh PJ, Ul-Hasan S, van der Hooft JJJ, Vargas F, Vázquez-Baeza Y, Vogtmann E, von Hippel M, Walters W, Wan Y, Wang M, Warren J, Weber KC, Williamson CHD, Willis AD, Xu ZZ, Zaneveld JR, Zhang Y, Zhu Q, Knight R, Caporaso JG. Reproducible, interactive, scalable and extensible microbiome data science using QIIME 2. Nat Biotechnol. 2019 Aug;37(8):852-857. doi: 10.1038/s41587-019-0209-9. Erratum in: Nat Biotechnol. 2019 Sep;37(9):1091. PMID: 31341288; PMCID: PMC7015180. [↑](#endnote-ref-25)
26. Lang T. Documenting research in scientific articles: guidelines for authors: 2. Reporting hypothesis tests. Chest. 2007 Jan;131(1):317-9. doi: 10.1378/chest.06-2087. PMID: 17218594. [↑](#endnote-ref-26)
27. Ornish D, Brown SE, Scherwitz LW, Billings JH, Armstrong WT, Ports TA, McLanahan SM, Kirkeeide RL, Brand RJ, Gould KL. Can lifestyle changes reverse coronary heart disease? The Lifestyle Heart Trial. Lancet. 1990 Jul 21;336(8708):129-33. doi: 10.1016/0140-6736(90)91656-u. PMID: 1973470. [↑](#endnote-ref-27)
28. Ornish D, Weidner G, Fair WR, Marlin R, Pettengill EB, Raisin CJ, Dunn-Emke S, Crutchfield L, Jacobs FN, Barnard RJ, Aronson WJ, McCormac P, McKnight DJ, Fein JD, Dnistrian AM, Weinstein J, Ngo TH, Mendell NR, Carroll PR. Intensive lifestyle changes may affect the progression of prostate cancer. J Urol. 2005 Sep;174(3):1065-9; discussion 1069-70. doi: 10.1097/01.ju.0000169487.49018.73. PMID: 16094059. [↑](#endnote-ref-28)
29. Ornish D, Lin J, Chan JM, Epel E, Kemp C, Weidner G, Marlin R, Frenda SJ, Magbanua MJM, Daubenmier J, Estay I, Hills NK, Chainani-Wu N, Carroll PR, Blackburn EH. Effect of comprehensive lifestyle changes on telomerase activity and telomere length in men with biopsy-proven low-risk prostate cancer: 5-year follow-up of a descriptive pilot study. Lancet Oncol. 2013 Oct;14(11):1112-1120. doi: 10.1016/S1470-2045(13)70366-8. Epub 2013 Sep 17. PMID: 24051140. [↑](#endnote-ref-29)
30. Rahman G, Morton JT, Martino C, Sepich-Poore GD, Allaband C, Guccione C, Chen Y, Hakim D, Estaki M, Knight R. BIRDMAn: A Bayesian differential abundance framework that enables robust inference of host-microbe associations. bioRxiv [Preprint]. 2023 Feb 2:2023.01.30.526328. doi: 10.1101/2023.01.30.526328. PMID: 36778470; PMCID: PMC9915500. [↑](#endnote-ref-30)
31. Khedr EM, Omeran N, Karam-Allah Ramadan H, Ahmed GK, Abdelwarith AM. Alteration of Gut Microbiota in Alzheimer's Disease and Their Relation to the Cognitive Impairment. J Alzheimers Dis. 2022;88(3):1103-1114. doi: 10.3233/JAD-220176. PMID: 35754271. [↑](#endnote-ref-31)
32. Borsom EM, Conn K, Keefe CR, Herman C, Orsini GM, Hirsch AH, Palma Avila M, Testo G, Jaramillo SA, Bolyen E, Lee K, Caporaso JG, Cope EK. Predicting Neurodegenerative Disease Using Prepathology Gut Microbiota Composition: a Longitudinal Study in Mice Modeling Alzheimer's Disease Pathologies. Microbiol Spectr. 2023 Mar 6;11(2):e0345822. doi: 10.1128/spectrum.03458-22. Epub ahead of print. PMID: 36877047; PMCID: PMC10101110.

    Bottom of Form [↑](#endnote-ref-32)
